# Bridging model and experiment in systems neuroscience with Cleo: the Closed-Loop, Electrophysiology, and Optophysiology simulation testbed

**DOI:** 10.1101/2023.01.27.525963

**Published:** 2024-07-09

**Authors:** Kyle A. Johnsen, Nathanael A. Cruzado, Zachary C. Menard, Adam A. Willats, Adam S. Charles, Jeffrey E. Markowitz, Christopher J. Rozell

**Affiliations:** a Coulter Department of Biomedical Engineering, Georgia Institute of Technology and Emory University, Atlanta, GA, USA; b School of Electrical and Computer Engineering, Georgia Institute of Technology, Atlanta, GA, USA; c School of Physics, Georgia Institute of Technology, Atlanta, GA, USA; d Department of Biomedical Engineering, The Johns Hopkins University, Baltimore, MD, USA

## Abstract

Systems neuroscience has experienced an explosion of new tools for reading and writing neural activity, enabling exciting new experiments such as all-optical or closed-loop control that effect powerful causal interventions. At the same time, improved computational models are capable of reproducing behavior and neural activity with increasing fidelity. Unfortunately, these advances have drastically increased the complexity of integrating different lines of research, resulting in the missed opportunities and untapped potential of suboptimal experiments. Experiment simulation can help bridge this gap, allowing model and experiment to better inform each other by providing a low-cost testbed for experiment design, model validation, and methods engineering. Specifically, this can be achieved by incorporating the simulation of the experimental interface into our models, but no existing tool integrates optogenetics, two-photon calcium imaging, electrode recording, and flexible closed-loop processing with neural population simulations. To address this need, we have developed Cleo: the Closed-Loop, Electrophysiology, and Optophysiology experiment simulation testbed. Cleo is a Python package enabling injection of recording and stimulation devices as well as closed-loop control with realistic latency into a Brian spiking neural network model. It is the only publicly available tool currently supporting two-photon and multi-opsin/wavelength optogenetics. To facilitate adoption and extension by the community, Cleo is open-source, modular, tested, and documented, and can export results to various data formats. Here we describe the design and features of Cleo, validate output of individual components and integrated experiments, and demonstrate its utility for advancing optogenetic techniques in prospective experiments using previously published systems neuroscience models.

## 1. Introduction

Systems neuroscience is currently undergoing a revolution fueled by advances in neural manipulation [1–6] and measurement [7–11] technologies as well as data analysis methods [12–16]. These have yielded unprecedented datasets and insights into network activity, as well as novel experimental paradigms such as direct closed-loop control of neural activity [17–29]. At the same time, models from cognitive, computational, and theoretical neuroscience have grown both in their computational power and their concordance with experimental data. While exciting, this explosion in the sophistication and quantity of experimental data, tools, and models has led to a considerable amount of missed opportunities and untapped potential.

**Figure 1:**
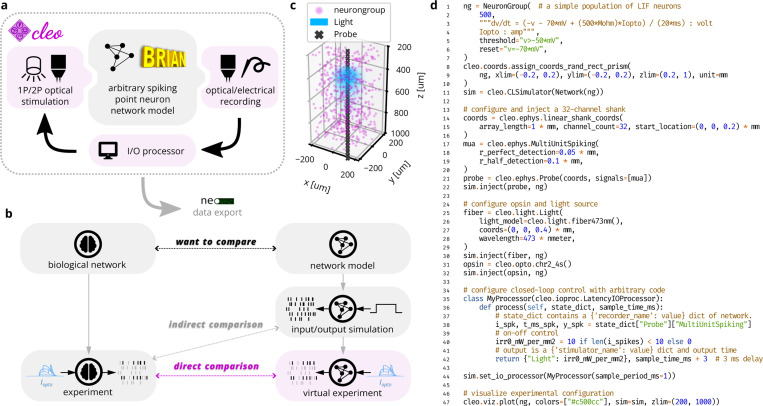
Cleo enables simulation of complex systems neuroscience experiments. (A) Cleo wraps a Brian network model, injects stimulation and recording devices, and interfaces with the network in real time through a simulated “I/O processor” to control stimulation devices in an optionally closed-loop and/or delayed fashion. Finally, results can be exported via the Neo Python package [30]. Pink shading indicates components provided by Cleo. (B) An illustration of Cleo’s utility as an experiment simulation testbed. By simulating the measurement and manipulation of the underlying neural activity, Cleo produces simulation results that are more directly comparable to electrophysiology experiments. This makes Cleo a valuable tool for experiment design, methods engineering, and model validation. (C) Graphical output (with slight modifications) of the example code in D. (D) Example code configuring a basic Cleo experiment. Note how few lines are needed to simulate multi-channel electrode recording, optogenetic stimulation, and delayed closed-loop control starting with a Brian Network model.

Building bridges between cutting-edge experiments and powerful models could enable these parallel lines of research to better inform and inspire each other. However, modern computational neuroscience models rarely account for the limitations imposed by measurement and manipulation tools, making it difficult to fully bridge between theory and experiment (see Fig. 1b) and simply impossible to adequately select from an ever-growing catalog of such tools via mental models or *ad hoc* design processes alone. Having a widely applicable framework for this type of integrated modeling informed by the constraints and idiosyncrasies of experimental interfaces would provide at least two benefits. First, this approach provides a testbed for low-cost, *in silico* prototyping of complex *in vivo* experiments, accelerating experiment design and the engineering of tools and techniques. This is especially important in closed-loop experiments, where real-time interaction with the neural system makes results harder to predict, and in experiments designed to adjudicate between multiple competing models accounting for prior observations. Second, because this approach facilitates the comparison of a computational model to experimental data, it enhances the model development process. For example, a modeler wishing to validate their results against data from a typical optogenetics/electrophysiology experiment can do so with greater confidence by simulating dynamic photocurrents and noisy spike detection than by simply injecting synthetic currents and perfectly recording every spike.

However, the increasing complexity of both experiments and models requires specialized software to meet this goal. While multiple existing tools facilitate some degree of stimulation and recording of high-level population simulations [31–36], these have significant limitations. Many are oriented towards detailed, multi-compartment neuron models that can be hard to develop or costly to run for large populations, and none offer a full suite of ready-to-use light, opsin, and imaging models for optophysiology. Moreover, none support flexible closed-loop control with the important feature of real-time processing latency, needed because of the aforementioned difficulty of predicting the impact design choices will have in feedback control experiments.

To address this crucial need, this paper describes the new open-source software Cleo: the Closed Loop, Electrophysiology, and Optophysiology experiment simulation testbed. Cleo integrates arbitrary closed-loop signal processing, recording, and stimulation devices that can be used in combination with existing Brian simulator [37] network models to simulate passive recording, open-loop stimulation, or closed-loop control experiments (see Fig. 1a). Cleo currently implements spike and approximate local field potential (LFP) recording, light and opsin models for one- and two-photon optogenetics, and two-photon calcium imaging, all with a modular design that allows for future addition of other modalities. We implement features tailored to point neuron models, though Cleo could be extended to support multi-compartment neurons in the future. For compatibility with existing analysis tools and pipelines, Cleo can also export simulation data via the Neo Python package [30], which in turn supports dozens of file formats. Here we describe the design and features of Cleo, and validate output both of individual system components and end-to-end experiments. We further demonstrate its utility in prospective experiments featuring a variety of use cases and existing models, including closed-loop inhibition of a traveling wave in sensory cortex, dynamic clamping of firing rate to disrupt visual cortex plasticity, and sharp wave-ripple evocation in the hippocampus.

## 2. Materials and Methods

### 2.1. Architecture and design rationale

In our design of Cleo, building an *in silico* experiment around an existing Brian spiking neural network model consists of (1) specifying the recording apparatus, (2) specifying the stimulation apparatus, and (3) configuring an I/O processor to control stimulation devices (see Fig. 1a,d). Cleo’s CLSimulator object integrates these components and orchestrates the experiment by injecting devices, running the Brian simulation, and communicating with an IOProcessor object at each time step. The IOProcessor receives measurements according to a user-specified sampling schedule and returns any updates to stimulator devices. Below, we describe the principles and assumptions that guided our modeling and software choices.

Two factors drove our choice of recording and stimulation models to integrate into Cleo. First, because Cleo’s purpose is to simulate experiments, we focused on models at the level of accessible experimental parameters. Because parameters such as electrode location, channel count, and optic fiber depth are all defined naturally in space, Cleo’s electrode, optogenetics, and imaging modules require a spatial network model where relevant neurons are assigned x, y, and z coordinates. Second, we tailored Cleo to systems neuroscience models that capture mesoscale phenomena (at the circuits/population level rather than single-cell or whole-brain levels) without high degrees of biophysical realism. Specifically, Cleo was developed primarily for point neuron rather than multi-compartment, morphological neuron models. While limiting the network model space compatible with Cleo, this choice dramatically simplifies software development and reduces simulation runtime, freeing researchers to move more quickly towards the ultimate goal of informed *in vivo* experiments. This decision had consequences in our software and modeling decisions (see Sec. 2.2, Sec. 2.3, Sec. 2.4).

In addition to our modeling priorities, the goals of usability, flexibility, and extensibility guided our choices in software dependencies and infrastructure. Ease of use is important to make Cleo as accessible as possible, especially to researchers with primarily experimental backgrounds. This usability goal also motivated Cleo’s modular design, which allows users to add different recording or stimulation devices with little or no modification to the underlying network model, easing the burden of testing a variety of experimental configurations (see Fig. 1c,d for example code and visualization). Flexibility in the underlying simulator, in addition to enabling compatibility with a wide variety of models, was a necessity for arbitrarily interacting with the simulation in a closed-loop fashion. Finally, we endeavored to make Cleo extensible so it could be adapted to use cases beyond the capabilities provided upon release, motivating the modular “plug-in” architecture that enables future incorporation of new experimental interfaces (e.g., microstimulation). In the following sections we describe the specific infrastructure and modeling choices we made in accordance with this rationale.

### 2.2. Simulator infrastructure

Other tools in the spirit of experiment simulation exist, though none with the collection of goals and functionality of Cleo. One is Mozaik [31], which can manage stimulation and recording parameters as well as data and visualizations, running on the simulator backend-agnostic PyNN interface [38]. It has been used to prototype and characterize advanced optogenetic control [39, 40], but PyNN does not provide an API for natively adding arbitrary differential equations to the core simulation (i.e., for features such as opsin and calcium dynamics). Three more (BioNet [34, 35], NetPyNE [36], and LFPy [41, 42]) include some of the features we needed, but as front-ends to the NEURON simulator [43] they are oriented towards biophysically detailed, expensive-to-simulate neuron models. The same can be said of VERTEX [32, 33], which is a tool for use in MATLAB. NAOMi [44] produces highly realistic two-photon calcium imaging data, but is not designed to capture other important facets of experiment simulation. See Table 1 for details.

Between the two most widely used spiking neural network simulators optimized for point neurons, Brian 2 (RRID:SCR_002998) [37] and NEST [45], we chose Brian for its intuitiveness and flexibility, following the example of other open-source projects [38, 46, 47]. It allows (and even requires) the user to define models mathematically rather than selecting from a pre-defined library of cell types and features, while maintaining the ease of a high-level interface. This keeps model and experiment details together and enabled us to define arbitrary recording and stimulation models that easily interface with the simulation. Moreover, Brian users only need to know Python: a programming language with the advantages of being open-source, intuitive to learn [48], and widely used in computational neuroscience [49, 50].

### 2.3. Optogenetics models

Cleo simulates optogenetic stimulation by combining a model of light propagation with an opsin model relating light to current. The light model is based on Kubelka-Munk light propagation, operating on the assumption that the medium is optically homogeneous and that particles are larger than the light wavelength [51, 52]. Cleo includes absorbance, scattering, and refraction parameters for 473-nm (blue) light as given in [51], but these are easily updated by the user for other wavelengths.

Independent of the light propagation model, Cleo provides two different opsin models. One is a four-state Markov model as presented in [46]. This model captures rise, peak, plateau, and fall dynamics of the photocurrent as opsins are activated and deactivated through a Markov process. By defining conductance rather than current directly, this model is also able to reproduce the photocurrent’s dependence on the membrane potential (see Fig. 3). While the four-state model fits experimental data fairly well, the code is structured so that three- or six-state models could also be easily implemented. Cleo provides parameters for channelrhodopsin-2 (ChR2) [53], ChR2(H134R) [54], Chrimson [55], Vf-Chrimson [56], GtACR2 [57], and eNpHR3.0 [58], as given by Evans *et al.* [46] and Bansal *et al.* [59]. Users wanting to take advantage of additional optogenetic innovations such as improved channel rhodopsins [3, 60–63], chloride pumps [64, 65] and channels [66], and others [65, 67] will need to provide opsin model parameters, many of which are available in published literature [59, 68–71].

However, because the Markov model depends on somewhat realistic membrane potential and resistance values, it is not well suited for many pre-existing models that do not. For example, many commonly used leaky integrate-and-fire (LIF) neurons define the membrane potential as ranging from 0 to 1, rather than −70mV to −50 mV, rendering both the units and values (relative to the opsin’s reversal potential) incompatible. While one could adapt neuron models for compatibility with this Markov opsin model, to minimize user burden we also developed an alternative model that delivers photocurrent proportional to the light intensity at each neuron. Specifically, we offer an optional model of the opsin current described with

(1) $$I_{\text{opto}}=k*\text{Irr}*\rho_{\text{rel}}$$

where k is an arbitrary gain term, Irr is the irradiance of the light source at the location of the neuron with unit mW/mm^2^, and $\rho_{\text{rel}}\geq 0$ is the relative opsin expression level (the default value of 1 corresponding to the standard model fit). Note that k is expressed in $\text{[unit of}\;I_{\text{opto}}]*{\text{mm}}^{2}/\text{mW}$, adapting to the units of $I_{\text{opto}}$. This model allows users to retain their neuron model with parameters and units unchanged, since they can adapt the k term to whatever scale and units are needed. Preliminary experiments show that this simplified opsin model (see Extended Data Fig. 2) can produce responses that are similar in many respects to those of the four-state Markov model.

In addition to options for opsin modeling, Cleo allows the user to specify both the probability that cells of a target population express an opsin and the per-cell expression level (via the afore-mentioned $\rho_{\text{rel}}$ parameter). Users can thus study the impact of heterogeneous opsin expression on the outcome of an experiment. We note that this model does not describe long-term decay in opsin efficacy with prolonged stimulation.

#### 2.3.1. Multi-wavelength sensitivity

More sophisticated experimental manipulations may require the use of multiple opsins simultaneously. However, overlapping wavelength sensitivities can lead to crosstalk; i.e., a given opsin pair may not be independently controllable when light at one wavelength activates both opsins. Cleo simulates this important phenomenon using the action spectrum of each opsin. We extracted action spectra from literature [53, 56–58] and represented the normalized response for stimulation of given irradiance with the factor $\varepsilon \left({\text{λ}}_{\text{other}}\right)$ [59]. For an opsin receiving light from two wavelengths, ${\text{λ}}_{\text{peak}}$ and ${\text{λ}}_{\text{other}}$, we then compute the effective irradiance for a given neuron as

(2) $${\text{Irr}}_{\text{eff}}{\text{=Irr}}_{{\text{λ}}_{\text{peak}}}+\varepsilon \left({\text{λ}}_{\text{other}}\right){\text{Irr}}_{{\text{λ}}_{\text{other}}}.$$

Combining irradiance linearly between light source makes the simplifying assumption that an opsin’s response to photostimulation is a linear function of irradiance (see Supplemental Information for details). For an example simulation of multi-wavelength, multi-opsin stimulation, see Extended Data Fig. 3.

### 2.4. Electrode recording models

Because we have prioritized point neuron simulations, the electrode functionality currently implemented in Cleo does not rely on biophysical forward modeling of extracellular potentials that could only be computed from multi-compartment neurons [72, 73].

#### 2.4.1. Spiking

**Figure 2:**
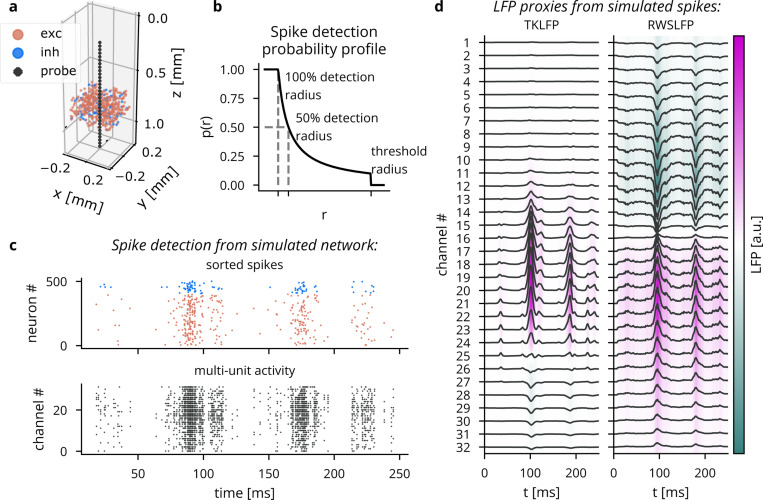
Illustration of LFP and spiking from Cleo’s electrophysiology module in a simulated excitatory/inhibitory network. (A) A plot generated by Cleo showing the positions of neurons and electrode contacts. The contacts emulate a 32-channel linear NeuroNexus array. (B) The probabilistic spike detection model. All spikes within the 100% detection radius, 50% of spikes at the 50% detection radius, and none of those outside the threshold radius are recorded. The detection probability decays with 1/r. (C) Spiking activity recorded in the setup shown in A. Top: the sorted spike signal, which gives the ground truth source neuron for every spike as a perfect proxy for spike sorting. Bottom: multi-unit activity, where spikes are reported on every channel they are detected on, regardless of the source neuron. (D) The two LFP proxy signals provided by Cleo, recorded from the same simulated network/activity in A/C.

To approximate spike recording without filtering and thresholding of extracellular potentials, Cleo captures ground-truth spikes (returned by the Brian simulator) and stochastically determines which to report as recorded on the electrode. The probability a given spike is detected by an electrode is a function of r, the distance between the neuron and the electrode. This function is parametrized by a perfect detection radius (where all spikes are reported), a half detection radius (where there is a 50% chance a spike will be detected), and a cutoff radius (where all neurons are ignored). The detection probability function is interpolated between the parametrized points with a 1/r function [74] (see Fig. 2b). The user may refer to studies such as [75] to determine reasonable detection distance parameters.

Cleo provides spike recording functionality in two forms: multi-unit and sorted (see Fig. 2c). Multi-unit activity reports every spike detected by every channel, without regard for the origin of the spike. Thus, each channel can report spikes from multiple neurons and a single spike can be reported on multiple channels. Sorted spiking, on the other hand, reports all spikes detected on at least one channel, where each neuron is identified by a unique index. Because point neurons cannot provide the raw extracellular potential waveforms needed for spike sorting algorithms, we approximate the spike sorting process by assuming perfect sorting. While real-time spike sorting is currently not feasible in practice for large channel counts, this sorted spiking option could be used to emulate a workflow of isolating one or a few neurons to record spikes from in real time.

#### 2.4.2. LFP

To approximate cortical LFP without resorting to morphological neurons and biophysical forward modeling, we implemented two LFP proxy signals that con be computed from point neuron simulations.

The first approximates the per-spike contribution to LFP with a delayed Gaussian kernel, where amplitude and delay depend on the position of the neuron relative to the electrode, as well as cell type (excitatory or inhibitory) [76] (see Fig. 2d). We hereafter refer to this proxy signal as Teleńczuk kernel LFP (TKLFP). Default parameters (taken from the original study) were estimated from human temporal cortex experimental data and from hippocampus simulations. As the authors indicate, parameters may need refinement on a per-region basis. While the original study included reference peak amplitude ($A_{0}$) values at just four cortical depths, we inferred these values for arbitrary depths by performing cubic interpolation on reported data (see Figure 5 in [76]) and assumed that this profile dropped to zero at 600 pm below the soma and 1000 pm above.

Cleo also provides the Reference Weighted Sum of postsynaptic currents LFP proxy (RWSLFP) [77], which fits the forward model LFP well ($R^{2}>0.9$) for standard pyramidal cell morphologies when network activity and recording location yield a sufficiently large signal. This method sums AMPA and GABA currents onto pyramidal cells, each current with a different weight and time delay. The amplitude of the signal is then determined by the axial and lateral recording distances, relative to pyramidal cells’ apical dendrites. To support arbitrary recording locations, we interpolated and extrapolated this amplitude profile as given in Figure 2B of the original publication. We did this by fitting a scaled beta distribution kernel at each radial distance and interpolating linearly between these fits. Because these signal amplitudes were evaluated by summing currents over a population distributed within a 250 pm-radius cylinder, Cleo supports arbitrary morphologies by providing an alternate amplitude profile optimally scaled such that the sum of individual neurons’ contributions is close to the population profile. We also include a scaled version of the closed-form per-neuron contribution as given by Aussel *et al.* [78].

A major difference between the two methods is that TKLFP is computed from spikes alone, while RWSLFP requires synaptic currents. Continuing in the spirit of supporting simplistic network models, Cleo provides the option to synthesize synaptic currents instead of simulating their dynamics by convolving spikes with a biexponential kernel (see Eq. (5.34) in [79]), requiring only that the user specify which synapses mediate these spikes. The basis in currents allows RWSLFP to better capture high-frequency signals deriving from subthreshold activity (see Extended Data Fig. 10).

As there was no publicly available code implementing these methods, we created, tested, and documented standalone implementations in the tklfp and wslfp Python packages [80, 81]. The authors’ goal of lowering the cost of LFP simulation is thus aided as their methods are easily accessible for the first time, for use inside or outside Cleo.

### 2.5. All-optical control

#### 2.5.1. Two-photon microscopy

Cleo simulates microscopy by taking microscope location, image width, focus depth, and soma size, and selecting neurons with a cross section in the plane of imaging. Calcium traces are generated for the given regions of interest (ROIs), adding Gaussian noise of standard deviation $\sigma_{\text{noise}}$ that depends both on the indicator and on the size of the soma cross section in focus. We model noise as Gaussian as a consequence of the central limit theorem, since the ROI measurement is a sum of per-pixel stochastic measurements [82| (see Extended Data Fig. 6). Accordingly, we scale $\sigma_{\text{noise}}$ with $1/\sqrt{N}$, where N is the number of visible pixels relative to the maximum (when the center of the soma lies exactly on the focal plane; see Fig. 5b,c, Extended Data Fig. 7). Signal strength is proportional to expression, denoted as $\rho_{\text{rel}}$ as with opsins (see Fig. 5c). Thus, for ROI i:

(3) $${\text{SNR}}_{\text{indicator}}=\frac{\text{Δ}F/F_{0_{1\text{AP}}}}{\sigma_{\text{noise}}}$$

(4) $${\text{SNR}}_{i}={\text{SNR}}_{\text{indicator}}\frac{\rho_{{\text{rel}}_{i}}}{1/\sqrt{N_{i}}}\propto \rho_{{\text{rel}}_{i}}\sqrt{N_{i}},$$

where $\text{Δ}F/F_{0_{1\text{AP}}}$ is the $\text{Δ}F/F_{0}$ peak after a single action potential. $\text{Δ}F/F_{0_{1\text{AP}}}$ and $\sigma_{\text{noise}}$ are indicator-specific and taken from Dana *et al.* [83] and Zhang *et al.* [84]. ROIs with signal-to-noise ratio (SNR) above a specified cutoff are selected for recording.

#### 2.5.2. Calcium indicator model

Cleo simulates intracellular calcium concentration dynamics using a biophysical model described previously in literature [44, 82, 85]:

(5) $$\frac{\text{d}\left[{\text{Ca}}^{2+}\right]}{\text{d}t}=-\text{γ}\frac{\left[{\text{Ca}}^{2+}\right]-{\left[{\text{Ca}}^{2+}\right]}_{\text{rest}}}{1+\kappa_{S}+\kappa_{B}}$$

(6) $$\text{Δ}\left[{\text{Ca}}^{2+}\right]\left(t_{\text{spike}}\right)=\frac{\text{Δ}{\left[{\text{Ca}}^{2+}\right]}_{T}}{1+\kappa_{S}+\kappa_{B}}$$

(7) $$\kappa_{B}=\frac{{\left[\text{B}\right]}_{T}K_{d}}{{\left(\left[{\text{Ca}}^{2+}\right]+K_{d}\right)}^{2}},$$

where γ is the clearance rate, $\kappa_{S}$ is the endogenous ${\text{Ca}}^{2+}$ binding ratio, $\kappa_{B}$ is the ${\text{Ca}}^{2+}$ binding ratio of the exogenous buffer (the indicator), $K_{d}$ is the indicator dissociation constant, ${\left[\text{B}\right]}_{T}$ is the total intracellular indicator concentration, and Δ ${\left[{\text{Ca}}^{2+}\right]}_{T}$ is total [${\text{Ca}}^{2+}$] increase per spike. Following Song et al. [44], [${\text{Ca}}^{2+}$] (t) is then convolved with a double exponential curve h(t) to obtain $\left[{\text{CaB}}_{\text{active}}\right]$, reflecting the response kinetics (such as binding and activation) not accounted for by binding affinity $K_{d}$ alone [86]:

(8) $$h(t)=A\left(1-e^{-t/\tau_{\text{on}}}\right)e^{-t/\tau_{\text{off}}}$$

(9) $$b(t)=\left(\left[{\text{Ca}}^{2+}\right](t)-{\left[{\text{Ca}}^{2+}\right]}_{\text{rest}}\right)*h(t)$$

(10) $$\left[{\text{CaB}}_{\text{active}}\right](t)=b(t)+{\left[{\text{Ca}}^{2+}\right]}_{\text{rest}}.$$

Parameters A, $\tau_{\text{on}}$, $\tau_{\text{off}}$ are indicator-specific. This convolution is approximated as integration of an ODE for ease of simulation (see Supplemental Information).

$\text{Δ}F/F_{0}$ is then computed from $\left[{\text{CaB}}_{\text{active}}\right]$ using a Hill equation nonlinearity and subtracting the baseline value to produce $\text{Δ}F/F_{0}=0$ when $\left[{\text{CaB}}_{\text{active}}\right]={\left[{\text{Ca}}^{2+}\right]}_{\text{rest}}$:

(11) $$\text{Δ}F/F_{0}=\text{Δ}F/F_{0_{\max}}\left(\frac{1}{1+{\left(K_{d}/\left[{\text{CaB}}_{\text{active}}\right]\right)}^{n_{H}}}-\frac{1}{1+{\left(K_{d}/{\left[{\text{Ca}}^{2+}\right]}_{\text{rest}}\right)}^{n_{H}}}\right).$$

Parameter values for various genetically encoded calcium indicators (GECIs) are taken from the NAOMi simulator [44]. For an example of simulated traces, see Extended Data Fig. 4c.

#### 2.5.3. Two-photon photostimulation

Cleo simulates two-photon (2P) photostimulation using the same opsin models previously described (Sec. 2.3) by modeling focused laser illumination. As is commonly reported in 2P experiments, laser power is used to define stimulation intensity. We convert from power to irradiance (needed for opsin models) by dividing by soma area [87], assuming a diameter of 20 pm. We then model off-target effects using a Gaussian ellipsoid point spread function with $\sigma_{\text{axial}}>\sigma_{\text{lateral}}$, as reported in literature [88–91] (see Fig. 5b). When targeting cells identified by the microscope, the laser is focused on the plane of imaging, such that the farther off-plane cells, the weaker they are stimulated. Morphological factors of 2P photostimulation such as membrane-boundedness of the opsin and differential expression between the soma and processes are not modeled.

### 2.6. Latency model

To simulate the effects of real-time compute latency, Cleo provides a LatencylOProcessor class capable of delivering control signals after arbitrary delays. It does this by storing the outputs calculated for every sample in a buffer along with the time they can be delivered to the network. For example, if a sample is taken at time t=20ms and the user wishes to simulate a 3 ms delay, the control signal and output time (23 ms) are stored in a buffer which the simulation checks at every time step. As soon as the simulation clock reaches 23 ms, the control signal is taken from the buffer and applied to update the stimulator devices. Because the user has arbitrary control over this latency, they can easily simulate the effects of closed-loop experimental constraints. For example, one could use probabilistic delays to assess the effect closed-loop algorithms with variable round-trip times between measurement and stimulation. By default, LatencyIOProcessor samples on a fixed schedule and simulates processing samples in parallel (i.e., the computation time for one sample does not affect that of others). This and the other sampling schemes Cleo provides are illustrated in Extended Data Fig. 8.

### 2.7. Neo export

To maximize compatibility with existing data analysis packages and pipelines, Cleo supports data export using Neo (RRID:SCR_000634), a Python package providing an in-memory representation of neuroscience data and read/write capabilities for dozens of file formats [30, 92]. Analysis code developed for experiments could thus be reused for simulated data, and vice versa.

### 2.8. Computing environment, performance, and code

Experiments (described in Sections 3.2 and 3.3) were run in one of two environments. The first is the Georgia Tech Partnership for Advanced Computing Environment (PACE) Phoenix cluster with 64 GB RAM, dual Intel Xeon Gold 6226 CPUs @ 2.7 GHz (24 cores/node), DDR4–2933 MHz DRAM, and Infiniband 100HDR interconnect. The second is a Dell consumer laptop with an Intel i9–9980HK CPU @ 2.40 GHz (8 cores) and 32 GB RAM. Code for experiments can be found at https://github.com/siplab-gt/cleo/tree/master/notebooks, https://github.com/siplab-gt/cleo-traveling-wave-rejection, https://github.com/siplab-gt/cleo-v1-plasticity-expt, and https://github.com/siplab-gt/cleo-hpc-experiments. See Table 1 for a list of the computing environment, Cleo version, and runtime of each experiment.

### 2.9. Feedback control

Validation experiment 1 used proportional-integral (PI) control and firing rate estimation as described in the original study [21] via an *ad hoc* implementation. Prospective experiment 2 used PI control and exponential firing rate estimation as described in [22]. Cleo provides implementations of these, which can be found in the cleo.ioproc module. Prospective experiment 3 used a standard linear quadratic regulator (LQR) approach as described in [23] and implemented in the ldsCtrlEst C++ library v0.8.1 [93]. ldsCtrlEst is part of CLOCTools [94, 95], a larger collection of algorithms and utilities for implementing closed-loop optogenetics in real-time lab experiments. Prospective experiment 3 also used a custom implementation of model-predictive control (MPC). We added 3 and 6 ms of latency to LQR and MPC, respectively, to simulate computation time. For details on model fitting and control parameters, see Supplemental Information.

## 3. Results

We demonstrate the utility of Cleo with a variety of different results. First, we validate output from the optogenetics and LFP recording modules by comparing to data from published literature. This confirms that these nontrivial models are suitable for integration into larger simulations. Next, to establish the validity of combining multiple models into the unified simulation of a complete experiment, we compare the results of three end-to-end validation experiments to published data for various experimental paradigms. Finally, we provide examples of how Cleo can be used to prototype novel closed-loop optogenetic techniques in three prospective experiments using previously published network models. Table 2 describes the runtime of each experiment.

### 3.1. Component validation

#### 3.1.1. Optogenetics model validation

**Extended Data Fig. 1:**
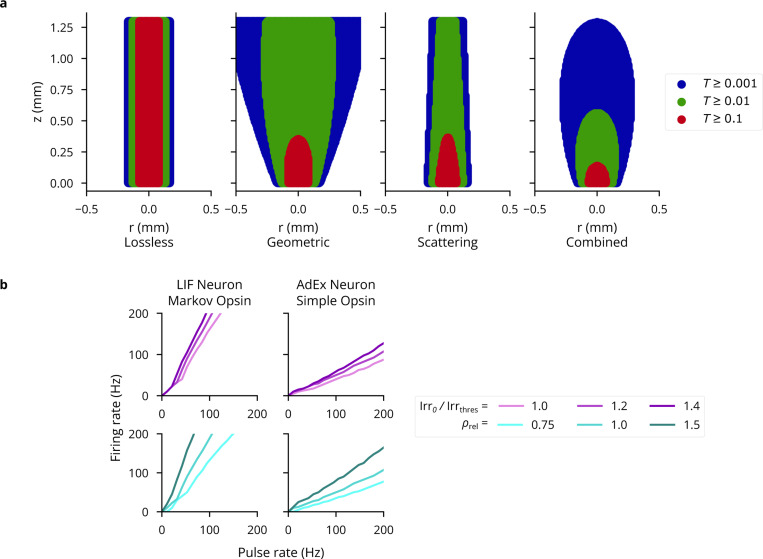
(A) Light transmittance T as a function of radius and axial distance from the optic fiber tip (cf. Figure 2a from [51]). The contribution of the Gaussian distribution, cone-shaped light propagation, and scattering are depicted separately. (B) Firing rate-pulse rate relationship as in Fig. 3c, for more neuron model-opsin combinations, namely LIF neuron with four-state Markov opsin and AdEx with a proportional current opsin. 5 ms pulses are used as before, with irradiance and expression levels as shown in the legend.

**Figure 3:**
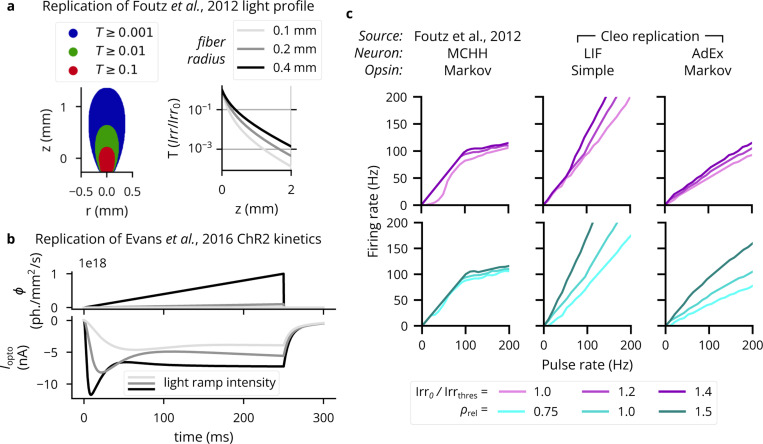
Validation of the optogenetics module. (A) Left: Light transmittance T as a function of radius and axial distance from the optic fiber tip (cf. Figure 2a from [51]). See Extended Data Fig. 1a for more detail. Right: Light transmittance T as a function of distance z straight out from the fiber tip for different optic fiber sizes (cf. Figure 2b from [51]). (B) Photocurrent $I_{\text{opto}}$ for ramping light of different intensities (cf. Figure 4c of [46]). (C) Neuron firing rates in response to optical stimulation with 5-ms pulse frequencies ranging from 1 to 200Hz. The left column re-plots data from [51]. The middle column shows results for an LIF neuron with a simple opsin, and the right column for a tonic AdEx neuron [96] with a Markov opsin model. The top row shows results for different light intensities: 100%, 120%, and 140% of the threshold for producing a single spike with a 5-ms pulse. The bottom row shows results for different expression levels relative to the default, $\rho_{\text{rel}}$. See Extended Data Fig. 1b for more neuron model-opsin combinations.

To validate Cleo’s light and opsin models, we first reproduced a previously reported optic fiber light transmission model [51]. The model defines transmittance T as the proportion of irradiance at a given point Irr to the irradiance at the fiber tip Irr_0_. Fig. 3 demonstrates this Cleo’s transmittance model corresponds to previously reported results as a function of radius and axial distance from the optic fiber tip (cf. panel A and Figure 2a of [51]) and distance z straight out from the fiber tip (cf. panel B and Figure 2b of [51]). See also Extended Data Fig. 1a. Validating the four-state opsin kinetics model, we also reproduced the ChR2 photocurrents in response to ramping light stimuli of varying intensities (cf. panel C and Figure 4c of [46]).

To test how well simplified models produce realistic firing patterns on long timescales, we also compared pulse rate to firing rate for a variety of light intensities and opsin expression levels (similar to previous studies with multi-compartment Hodgkin-Huxley neurons in [51]). We used combinations of leaky integrate-and-fire (LIF) and adaptive exponential integrate-and-fire (AdEx) [97] neuron models, along with proportional current and Markov opsin models. AdEx neurons had parameters as given by [96] for a tonic firing pattern, and irradiance was simulated at 120% of the single-spike threshold. As expected, the different model combinations behave differently and none reproduce exactly more detailed biophysically realistic simulations (see Fig. 3c, Extended Data Fig. 1b). Specifically, they reproduce the linear relationship at lower pulse rates and fail to capture the sublinear relationship at higher pulse rates, which could be remedied if desired through the inclusion of adaptive or refractory properties in the neuron model.

#### 3.1.2. LFP model validation

In addition to providing unit tests in the Cleo, wslfp, and tklfp codebases, we validated Cleo’s LFP output by comparing to previously published results. To test Cleo’s Teleńczuk kernel LFP approximation module, we reproduced the demo presented by [76] and found that Cleo’s output was essentially identical (see Extended Data Fig. 9a). We also compared TKLFP and RWSLFP output of the hippocampus model to its summed synaptic current LFP proxy and found them all to be qualitatively similar (see Sec. 3.3.3, Extended Data Fig. 9b). Here and in further comparisons (see Extended Data Fig. 10), we confirmed that TKLFP underrepresents high-frequency components compared to RWSLFP, as reported in the original publication. We also find its sign inverts at a depth other than that predicted by detailed biophysical modeling, namely, around the midpoint of pyramidal cell dipoles [77]. These evaluations suggest that the methods are implemented correctly and can thus be applied to a variety of modeling applications, subject to the limitations described by their authors.

### 3.2. End-to-end validation experiments

#### 3.2.1. Validation experiment 1: LFP recording of epileptiform hippocampus activity after Aussel et al.

**Figure 4:**
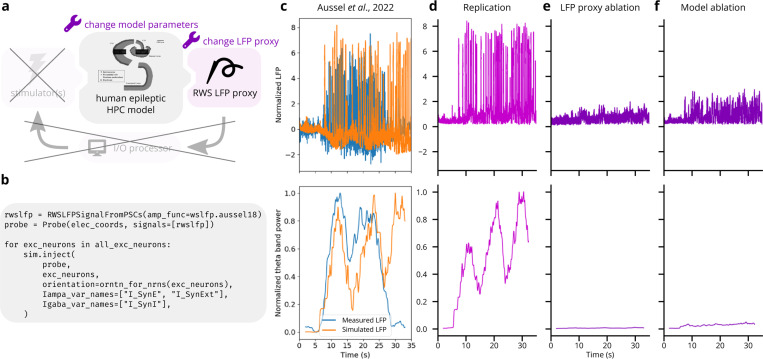
Reproduction of electrophysiological recordings of epileptiform hippocampus activity [78]. (A) Schematic of experiment setup. LFP is recorded from a hippocampal model [78], and ablations of both the model parameters and LFP output serve as negative controls. (B) Minimal code required to record LFP from the existing model. This replaces hundreds of lines in the original model code. (C) Top: Experimental and simulated LFP (estimated from summed synaptic currents). LFP is normalized to have a peak of 1 during the first 5 seconds of the simulation. Bottom: theta band power (see Supplemental Information for calculation details), normalized by the peak value. Image used under the CC BY 4.0 license. (D) Replication of C via Cleo’s RWSLFP recording. Theta power is normalized by the peak value. (E) Same as D, but with the average model input serving as an ablated LFP output. Theta power is normalized by the peak in D. (F) Same as D, but with model parameters corresponding to a healthy, rather than an epileptic state. Theta power is again normalized by the peak in D.

We illustrated Cleo’s utility in simulating electrophysiology experiments by replicating epileptiform activity recorded from the human hippocampus [78] (see Fig. 4a). We used the model described in [98], which delivers realistic inputs derived from stereoelectroencephalography (SEEG) recordings in three regions afferent to entorhinal cortex: the prefrontal cortex, the lateral temporal lobe, and the temporal pole. The authors show that when parameters are tuned to unhealthy states, the model exhibits epileptiform activity matching the SEEG data (see Fig. 4c). We wrapped this model with Cleo, delivered the inputs derived from afferent brain area recordings, and recorded LFP with electrodes in the same location as in the experiment. Cleo’s LFP output clearly reproduces the epileptiform activity present in the data, suggesting Cleo can usefully simulate electrophysiology experiments provided a satisfactory spiking neural network model (see Fig. 4d). We also ran the same simulation with ablations of LFP recording (using the average SEEG input instead of RWSLFP as the proxy signal) and the model (using healthy rather than epileptic model parameters) to evaluate the strength of this result. These ablations (see Fig. 4e,f) failed to produce the heightened signal and theta band power seen in the original data, suggesting that the accuracy of both the source model and the RWSLFP proxy method play a nontrivial role in replicating the experiment.

#### 3.2.2. Validation experiment 2: All-optical stimulation and recording of individual neurons after Rickgauer et al.

**Figure 5:**
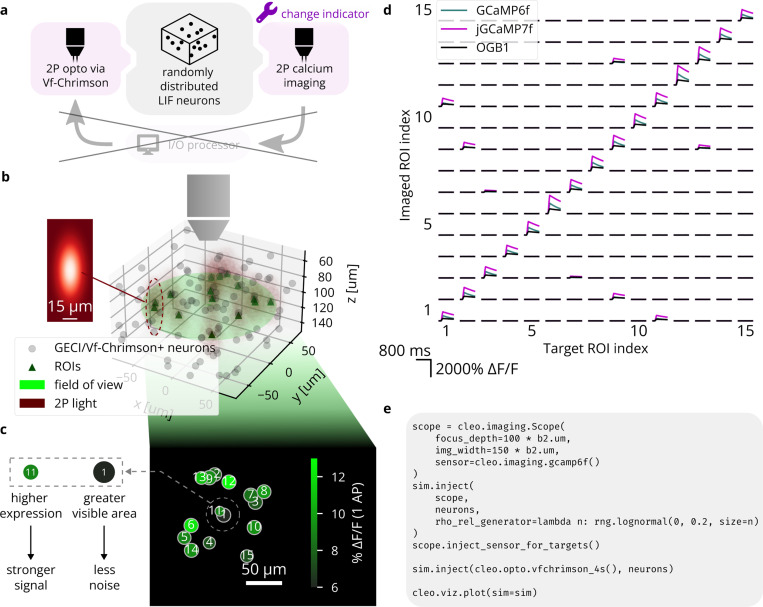
Reproduction of an end-to-end all-optical control experiment, after Figure 3 of [89]. (A) A schematic of the experiment configuration. Different calcium indicators are simulated to demonstrate Cleo’s capability to aide experimental design. (B) A 3D plot of the model spiking neural network with the microscope’s field of view visualized. Dark red ellipsoids depict laser light intensity around targeted neurons. *Inset:* A heatmap visualization of the Gaussian point spread function defining light intensity around each 2P stimulation target; cf. Figure 3b of [89]. The x and y axes correspond to lateral and axial axes, respectively. (C) 2D image as seen by the microscope; cf. Figure 3c of [89]. Size represents how much of each ROI is visible, i.e., how well centered it is on the focal plane. Color indicates signal strength, as determined by expression levels. (D) Results from the simulated all-optical experiment; cf. Figure 3c of [89]. Microscopy and photostimulation are configured as in B, performing calcium imaging using a model of the OGB-1, GCaMP6f, and jGCaMP7 indicators [83, 99, 100]. Each ROI is targeted one at a time (represented in each column), receiving 10 pulses of 2 ms width at 100 Hz. The recorded calcium trace of each ROI is shown in each row. Off-target effects can be seen for neurons that are close together (6 and 20, 18 and 19). (E) Minimal code example for configuring all-optical control, including the microscope, opsin, and calcium indicator. rho_rel refers to the expression level.

To validate Cleo’s simulation of two-photon, all-optical stimulation and recording, we reproduced the data presented in Figure 3 of [89], where individual neurons are controlled. Target LIF neurons with above-threshold SNR were chosen from a simulated population distributed randomly in 3D space. More modern (except in the case of OGB-1) molecular tools available in Cleo are substituted for the original GCaMP3/C1V1 indicator/opsin setup. One of three calcium indicators (OGB-1, GCaMP6f, or jGCaMP7 [83, 99, 100]) and the Vf-Chrimson opsin [56] were injected and each neuron was stimulated with 10 pulses of 2 ms width at 100Hz. 1060nm light at 2.5mW power was used for stimulation, assuming $\varepsilon_{\text{Vf-Chrimson}}$ (1060 nm) = 0.01. The resulting calcium traces in Fig. 5d reproduce the most important qualities of Rickgauer *et al*., Figure 3 [89], namely heterogeneity in signal and noise strength and independent stimulation of neurons, limited by spatial proximity. With regards to the latter, we see off-target ROIs respond significantly but more weakly than nearby targeted ROIs, as expected.

#### 3.2.3. Validation experiment 3: In vitro optoclamp after Newman et al.

**Figure 6:**
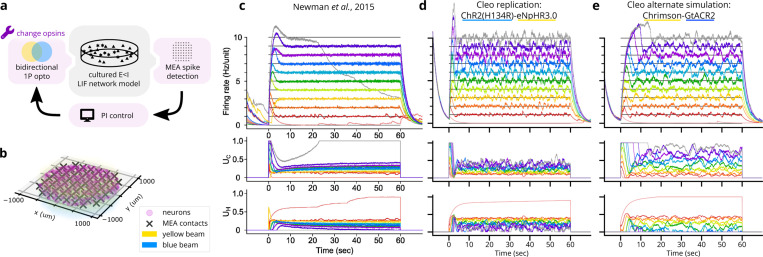
Reproduction of an end-to-end optogenetic feedback control (“optoclamp”) experiment [21]. (A) Schematic of the experimental setup. (B) 3D plot of network model, multi-electrode array, and light configuration. (C) Experimental data from Figure 2A of [21], showing firing rate (top), ChR2(H134R) control signal ($U_{C}$, middle), and eNpHR3.0 control signal ($U_{H}$, bottom) for each of 11 target firing rates, each marked with a different color. Image used under the CC BY 4.0 license. (D) Replication of B in a Cleo simulation. (E) Same as D, but with the Chrimson-GtACR2 opsin pair instead.

Demonstrating Cleo’s ability to capture salient features of closed-loop optogenetic control experiments, we reproduced the “optoclamp” experiment of [21] on cultured neurons. We simulated an E/I leaky-integrate-and-fire (LIF) network [101] of 800 excitatory and 200 inhibitory cells randomly distributed in a 2 mm diameter disc. Inhibitory weights were tuned to overpower excitatory weights, creating a network-wide bursting behavior. The multi-electrode array (MEA) had 60 contacts distributed with 200 pm as depicted in [21] and was configured to produce sorted spikes in real time. ChR2(H134R) [54] and eNpHR3.0 [58] were used as the excitatory and inhibitory opsins, respectively, injected with lognormal-distributed expression levels. These were targeted with uniform 465nm and 590nm light, respectively (see Fig. 6a). A proportional-integral (PI) controller as described in [21] determined these light levels to clamp firing rates to different target values. Detailed parameters can be found in the code repository. Our simulation (see Fig. 6c) reproduces key features of the experimental data (Fig. 6b) such as the initial overshoot/settling phase and the controller’s successful clamping of firing rate. Finer details such as post-inhibition rebound and the increase in required stimulation over time were not reproduced, highlighting how a Cleo simulation’s realism is limited by the SNN model provided. In this case, the simple E/I LIF network lacked adaptive or homeostatic mechanisms, and a number of parameters such as synaptic weights and opsin expression levels were not finely tuned.

We also ran the experiment with an alternate opsin pair, Chrimson [56] and GtACR2 [57], which required tuning a number of parameters differently to achieve similar results. The blue light was set to 450 nm wavelength to minimize activation of Chrimson, but control gains still needed to be adjusted to prevent Chrimson from overpowering GtACR2. Pulse frequency also needed to be increased to enable Chrimson to drive firing activity fast enough for higher target rates, presumably due to its faster off-kinetics. This process provides a glimpse into the difficulties of tuning closed-loop stimulation and shows how Cleo could be used to help design robust experiments.

### 3.3. Prospective experiments

#### 3.3.1. Prospective experiment 1: Closed-loop rejection of an S1 traveling wave

We implemented a rodent primary somatosensory cortex (S1) traveling wave model [102] in Brian to demonstrate Cleo’s capabilities for simulating an event-triggered closed-loop control experiment. The rodent S1 model uses a mix of excitatory and inhibitory neurons (12,500 total) with weak local connections and a sparse sub-network with stronger connections. The neurons lie in a 5 mm × 5 mm sheet, and we adjusted the initial state and input of the center 1 mm^2^-diameter circle to produce a sparse traveling wave of spreading activation in response to an initial stimulus as reported in the original publication. We altered the original model to use Euclidean distance rather than Manhattan distance in determining connection probabilities.

**Figure 7:**
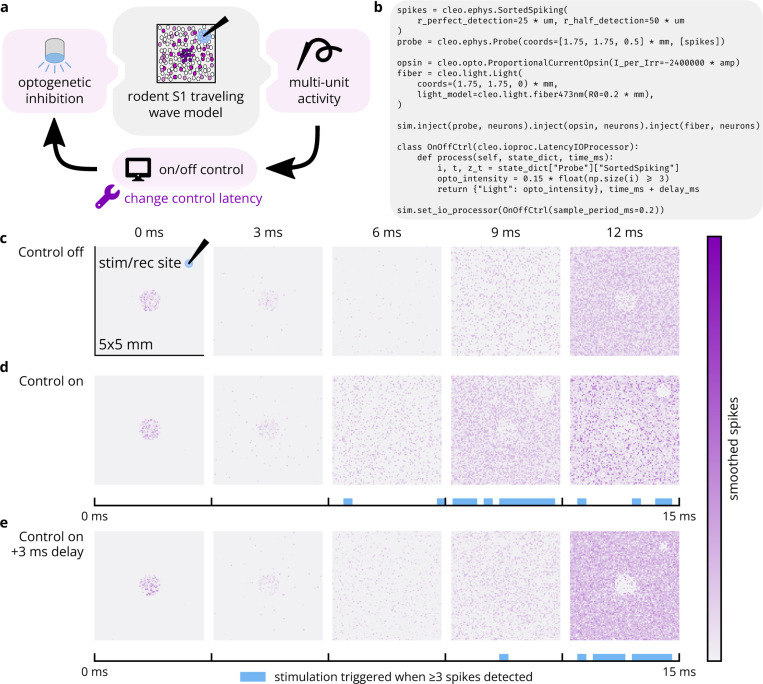
Cleo can simulate closed-loop inhibition of a whisker stimulation-evoked traveling wave. (A) Schematic of simulated experimental setup. The model consists of a 5 mm × 5 mm cortical area and optrode. The center 1mm^2^-diameter circle of neurons is strongly stimulated, initiating a traveling wave of activity radiating outward. When sufficient spiking is detected at the electrode, an optical stimulus activating an inhibitory opsin is triggered. (B) Minimal code sample to configure the non-model components of the experiment. (C) Spatial spiking rasters over time. Each pixel represents the firing rate of a neuron, smoothed with a Gaussian kernel of 0.8 ms. (D) Top: Results of another simulation as in C, but with closed-loop inhibition. Neural activity is clearly disrupted by the optogenetic stimulus in the neighborhood of the optrode. Bottom: Photostimulation over time. (E) Same as D, but with 3 ms latency introduced into the control loop. This latency clearly prevents the controller from rejecting the traveling wave as it first enters the vicinity of the optrode.

We configured Cleo to simulate an experiment with an “optrode” (a combined electrode and optic fiber) to trigger inhibitory optogenetic stimulation when recorded multi-unit activity reached 3 or more spikes over the previous 0.2 ms sampling period (illustrated in Fig. 7a). We used the previously described simple opsin model to accommodate the neuron model’s normalized, non-biophysical parameters and adjusted the optogenetic stimulus through trial and error to a level sufficient to suppress activity around the optic fiber. To assess the effect of control latency, we also simulated the same experiment with an added 3 ms delay. The model was run for 15 ms of simulated time.

As seen in Fig. 7c,d, the optogenetic stimulation suppresses neural activity, effectively quenching the traveling wave in the region around the optrode. As expected, delay in the control loop prevents effective suppression of the traveling wave as it first reaches the optrode (see Fig. 7e). This demonstrates the use of Cleo in simulating basic “reactive” or “event-triggered” control where a predetermined stimulus is presented in response to a detected feature in the electrophysiology recording. In general, this sort of closed-loop control might be used to either inhibit [26, 103] or amplify said feature. In this case, while constant inhibition could have achieved a similar effect, it would have posed a stronger intervention, increasing the likelihood of unnatural results. This prospective experiment also shows how Cleo can easily interface even with highly abstracted spiking neuron models.

#### 3.3.2. Prospective experiment 2: Clamping firing rate to disrupt plasticity in V1

**Figure 8:**
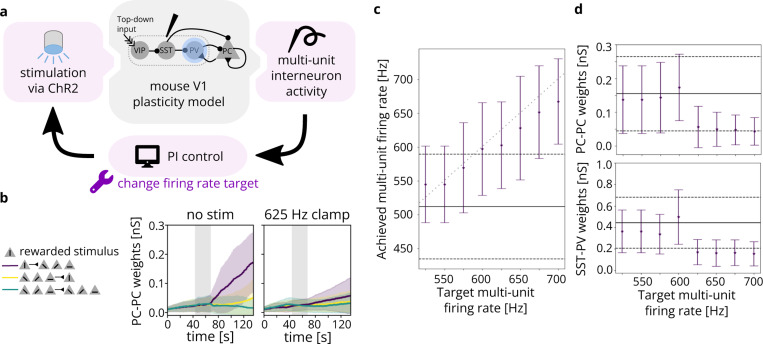
A Cleo simulation of optogenetic feedback control, clamping interneuron firing rate to disrupt top-down visual plasticity. (A) Schematic of the experimental setup. A model including simulated VIP, SST, PV, and PC neurons [104] was perturbed via optogenetic feedback control. The PI controller set light intensity targeting PV interneurons transfected with ChR2. (B) The neural weights across time for PC-PC connections. Neurons are grouped by which stimulus they were selective for, where the vertical stimulus was rewarded. PC-PC connection weights from neurons selective to the rewarded stimulus (S) to nonselective neurons (NS) are shown in purple, NS-S in yellow, and NS-NS in green. Mean weights are shown with solid lines and standard deviations are indicated by shaded regions. Top-down reward period is indicated by gray shading. Weights over time without (with) optogenetic control of firing rate are shown on the left (right). (C) Actual multi-unit, reward period firing rates for various targets. Dots indicate the mean over time; error bars are one s.d. Solid and dashed black lines indicate mean and s.d. for the unperturbed model. Gray dotted line marks where the target and detected firing rate are equal. (D) The weights at simulation end (t=126s) for PC-PC, S-NS connections (top) and for reward-selective SST-PV connections (bottom). Dots indicate the mean over synapses; error bars are one s.d. Solid and dotted black lines indicate mean and s.d. for the unperturbed model.

Feedback control promises the ability to more tightly control variables of interest, enabling stronger causal conclusions about their downstream effects. In this prospective experiment, for example, we demonstrate how a closed-loop controller simplifies obtaining a consistent, desired firing rate of a subset of neurons in a primary visual cortex (V1) layer 2/3 plasticity model [104], with the end of analyzing the effect on synaptic weight changes. A Brian 2 implementation of the model was publicly available on ModelDB [105] and required only the minor modification of assigning coordinates to neurons (random locations in a 400 μm × 400 μm × 200 μm volume). This model features a variety of neuron subtypes, including pools of vasoactive intestinal peptide-expressing (VIP), somatostatin-expressing (SST), parvalbumin-expressing (PV), and pyramidal (PC) cells. The network is defined with inhibitory connections VIP-SST, SST-PV, SST-PC, and PV-PC, as well as excitatory connections PC-PV and PC-PC (see Fig. 8a). A brief period (24.5 seconds) of top-down reward input to VIP is sufficient to cause substantial changes to neural weights in a longer post-reward period. This is because top-down reward causes SST to inhibit PV, which in turn disinhibits the PC, allowing for plasticity in the PC neurons that continues past the end of the reward period. Thus, we concluded that slightly disrupting PV activity should be sufficient to disrupt plasticity in the PC connections.

We used Cleo to model an electrode recording multi-unit inhibitory activity (spiking from SST and PV neurons), simulating the scenario where the cell type of incoming spikes is identified in real time based on their waveform. To establish a baseline, we observed spiking activity without any optogenetic stimulus, noting the mean and standard deviation of detected firing rate during the reward period to determine target firing rates for closed-loop control in subsequent simulations. Based on these results, we then ran 8 simulations, each with a different reference reward period firing rate ranging from 525 (just over the mean) to 700 (over one standard deviation above the mean) spikes per second. We followed the methods in [22], setting the light intensity in real time via a proportional-integral (PI) controller as implemented in the cleo.ioproc module. We used integral and proportional gains $K_{i}=0.003$ mW/mm^2^/spikes and $K_{p}=0.005$ mW/mm^2^/Hz. Firing rate was estimated via an exponential filter with time constant τ=1s. This model included a total of 694 neurons simulated over 137 seconds.

The resulting detected reward period firing rates for each target rate is shown in Fig. 8b. PI control modulated firing rates in predictable ways that agreed with the goals of the experiment. Specifically, the reward period firing rate clamp had clear effects on the weights of the neural connections, both for PC-PC connections and SST-PV connections (shown in Fig. 8c). High PV activity did indeed disrupt plasticity, reducing the weights for reward-selective synapses. The open-loop alternative to attain a given reference firing rate would be the careful and potentially time-consuming titration of stimulation levels. In this way, Cleo has demonstrated a nominal prototype of an experiment where closed-loop optogenetic control can potentially be used to draw a more compelling causal connection between components of a network. This also demonstrates Cleo’s built-in PI control algorithms which provide users with an easy point of entry to feedback control.

#### 3.3.3. Prospective experiment 3: Evoking SWRs in the hippocampus

To demonstrate Cleo’s capabilities to simulate optimal feedback control and approximate LFP, we interfaced Cleo with an anatomically informed model of the hippocampus previously described [78, 98]—the same used in Sec. 3.2.1. When a sustained external current is delivered to the entorhinal cortex of this model to simulate the slow waves of non-REM sleep, the model produces a sharp wave-ripple (SWR)-like pattern of LFPs, as approximated by summed synaptic currents. Our goal was to evoke a SWR using optogenetics in the absence of this strong square-wave input, illustrating how feedback control can reproduce a signal of interest at arbitrary times. Moreover, feedback control replaces a design and calibration process with model fit and controller tuning, producing a stimulation waveform that need not conform to a basic shape. In contrast, various experimenters have used rectangular, trapezoidal, and ramping pulses to optogenetically induce SWR-like oscillations *in vivo* that do not fully resemble spontaneous SWRs, apparently manually calibrating the intensity [106–108].

**Figure 9:**
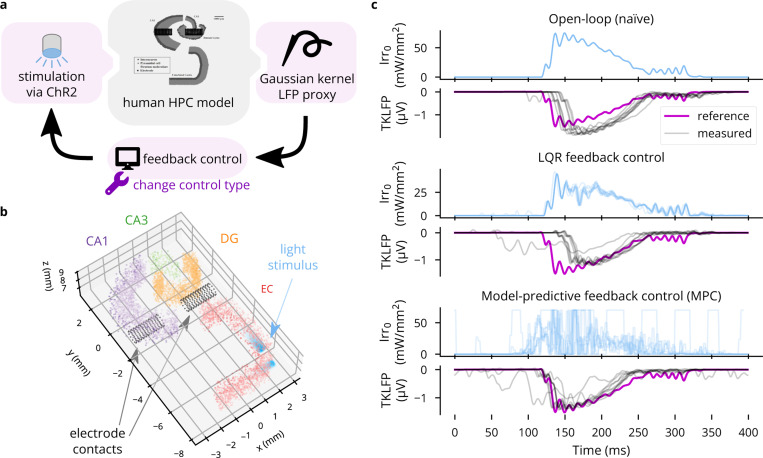
An example application of Cleo using optimal feedback control to follow a time-varying waveform. (A) Schematic of the simulated experiment setup. TKLFP is recorded from the anatomical hippocampus model by Aussel *et al*. [78, 98] and is fed into a feedback controller governing ChR2 photostimulation. (B) A 2.5 mm-thick slice of the 15 mm-tall model is shown. The model consists of four regions, entorhinal cortex (EC), dentate gyrus (DG), CA3, and CA1. Electrode contacts are represented as black dots and are in the same location as in the original model. Two light sources are shown in EC. Nine other such pairs (for a total of 20 light sources) not pictured here were spaced regularly parallel to the z axis. (C) Results are shown for ten trials each of three stimulation paradigms: naïve open-loop, LQR, and MPC. Input Irr_0_ is the light intensity at the tip of each optic fiber.

In the Cleo simulation, we placed simulated electrode contacts at the same locations as in the original model and used them to record LFP using the TKLFP approximation [76]. We then inserted optic fibers along the 15 mm model length and injected Gaussian process noise into the external current driving the model to create trial-to-trial variability. We illustrate three stimulation paradigms: naive open-loop (consisting of a mirrored, rectified version of the reference signal), linear quadratic regulator (LQR), and model-predictive control (MPC). The results demonstrate that Cleo can be used to simulate complex experimental scenarios with multiple recording and stimulation interfaces, and a variety of stimulation protocols can be prototyped on the same model with relative ease. In this case, the simulated response to stimulation is quite stereotypical, creating little meaningful trial-to-trial variation for the advantages of LQR over open-loop control to become apparent (see Fig. 9b). MPC, however, produces a notably earlier response than LQR since it is able to “look ahead”. The higher inter-trial variation in the stimulus waveform may also reflect the additional effort required to tune MPC or instability due to higher latency.

## 4. Discussion

Here we have presented Cleo, a Python package designed as a testbed for bridging point neuron spiking network models and experiments for mesoscale neuroscience. As the sole publicly available tool for simulating delayed closed-loop control, two-photon optogenetics, and multi-opsin/wavelength crosstalk, Cleo excels in consolidating various esoteric models into one adaptable platform, sparing researchers the need to understand and implement them on a case-by-case basis into their SNN simulations. By thus simulating the experimental apparatus, Cleo can bridge model and experiment by facilitating the process of model informing experiment and experiment informing model, which is a bidirectional research paradigm often advocated as providing the richest potential understanding of brain function.

A computational model can inform experiment as a substrate for a design and prototyping phase, which is important when considering advanced methods that require considerable time, resources, or risks to implement (especially true in closed-loop experiments). Thus, the researcher can answer beforehand questions such as whether an experiment is feasible [39], which opsin(s) or indicator(s) to use, what cells to target, where to record, or what closed-loop control algorithms perform adequately and with tolerable latency. By simulating the messy side effects of each choice, Cleo can help narrow down a number of suboptimal alternatives and make trade-offs between competing constraints. When a sufficiently realistic model for the studied system does not exist, multiple models representing possible variations in connectivity, parameters, or mechanisms could be used to cast light on which experimental configurations work best across hypothetical models. Indeed, the desired experiment in this case could be one that best adjudicates between these hypotheses [109].

Other potential applications of Cleo include facilitating the reverse process of experiment informing model. This is because Cleo can mimic the measurement and perturbation tools of modern systems neuroscience, producing results more directly comparable to experimental data than those of a synthetic input/ground-truth output simulation of the model. Subsequent analysis would allow the user to evaluate the model in the spirit of NeuronUnit [110], NetworkUnit [111, 112], and other such tools [113]. Yet another application of Cleo, in addition to aiding experiment design and model evaluation, is as a testbed for engineering hypothetical tools, helping answer questions such as, “What kinetics would be needed for a calcium indicator to effectively capture fast spiking interneuron activity?” or “What opsin kinetics would be needed to reproduce a complex temporal pattern in Purkinje cells?”

As mentioned previously, a primary motivation for developing Cleo was to accelerate the development of closed-loop optogenetic control (CLOC), which may enable stronger causal hypothesis testing. Neuroscientists have identified many network- and circuit-level variables and phenomena in search of interpretable explanations of brain activity. A natural application of CLOC is to control these features to enable stronger inference of their relationship to downstream variables. Examples of these potential targets for control include the activity of different cell types; the type [114], frequency [115], amplitude [115], spike coherence [116, 117] and interactions [118, 119] of different oscillatory patterns; discrete phenomena such as bursts, sharp wave-ripples, oscillatory bursts [120–122], traveling waves [123], or sleep spindles [124]; and latent states describing neural dynamics [125–128], including those most relevant to behavior [129, 130].

While some of these targets lend themselves easily to CLOC, others require continued innovation in interfacing technology. Specifically, stimulation technologies have been much more limited in their degrees of freedom than modern recording technology, and thus unlikely to sufficiently control many variables of interest. For this reason, the development of multi-channel micro-LED/optrode devices [6, 39, 131–141] and holographic optogenetic stimulation [3, 4, 87, 89, 90, 142] are of particular interest. Crucially, Cleo will enable rigorous investigation of both proposed specific technologies as well as general technological capabilities to guide new interface design.

While Cleo was designed to facilitate and accelerate the simulation of complex experiments as much as possible, it has several limitations. First, while Brian and Cleo have the flexibility to accommodate a wide variety of models, alternative tools and methods—adapted as necessary to simulate the experimental interface—may be better suited for larger spatiotemporal scales [35, 143, 144], higher levels of abstraction [145–147], and greater biophysical detail [34–36, 43]. A second limitation is that the flexibility that enables arbitrary closed-loop stimulation can slow down what might otherwise be a fast, purely compiled simulation.

Perhaps the biggest limitation is that the user must work to interface their model with Cleo, which could range from the simple task of assigning neuron coordinates to the considerable effort of re-implementing the model entirely with Brian, if not already a Brian model. Conversion from other simulators may be possible using the NeuroML [148] import feature, but its functionality is limited. Ideally, an experiment simulation testbed would flexibly support multiple simulation backends, as PyNN has provided for SNNs [38]. To do so in a native, computationally efficient way would require significant work, using the idiosyncrasies of each simulator to implement features they were not designed for (e.g., opsins, lights, and calcium indicators), as we have done for Brian. A future collaborative effort extending a multi-simulator framework such as PyNN for this purpose may be worth the investment if there is enough community interest in expanding the open-source SNN experiment simulation toolbox.

Cleo is open-source and can be installed from the Python Package Index under the name “cleosim”. The code can is hosted on GitHub at https://github.com/siplab-gt/cleo, where we invite users to submit feature requests, bug reports, pull requests, etc. Documentation, including an overview, tutorials, and API reference, can be found at https://cleosim.readthedocs.io. Future development of Cleo is relatively straightforward given Cleo’s modular structure. We anticipate future development to meet community needs may include simulation of different levels of abstraction (e.g., forward modeling of extracellular potentials [42, 72] for multi-compartment models or additional light propagation profiles [149]), additional/improved recording and stimulation modalities (e.g., photoelectric artifacts, voltage imaging, two-photon imaging/optogenetics crosstalk, electrical micro-stimulation, or an expanded selection of opsins and sensors), or support for heterogeneous sampling rates to capture scenarios such as when imaging is slower than electrode recording (similar to the approach taken by the real-time processing software Bonsai [150]).

## Extended Data

**Extended Data Fig. 2:**
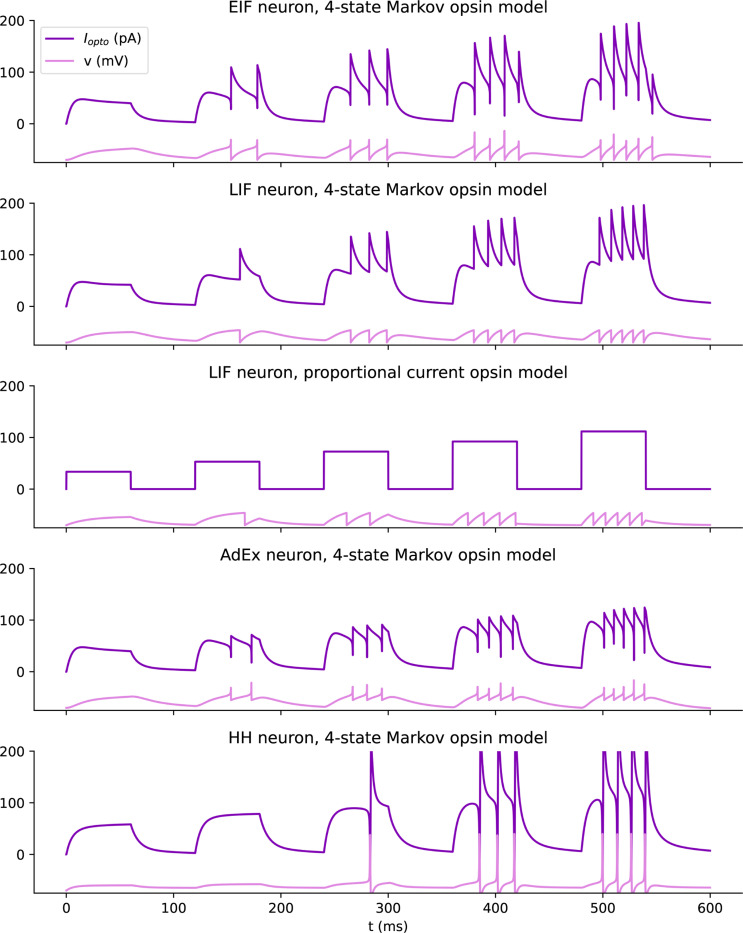
Comparison of different opsin and neuron model combinations, illustrating that qualitatively similar light-firing rate relationships can be achieved across a variety of model.

**Extended Data Fig. 3:**
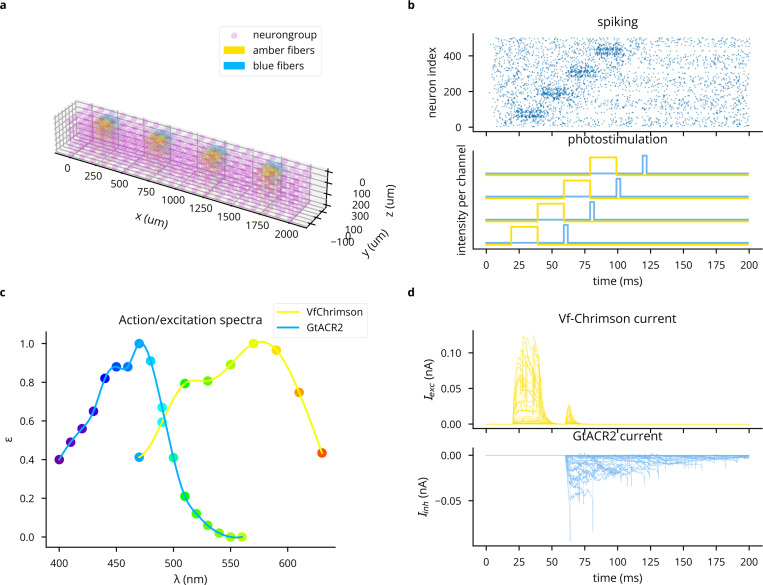
Demonstration of simulating multiple light sources, wavelengths, and opsins simultaneously. (A) 3D plot of network model and light sources. (B) Top: spike raster, where increasing neuron index correlates with increasing x coordinates. Bottom: Stimulation pattern for 473 and 590 nm light sources. (C) Action spectra of Vf-Chrimson and GtACR2, showing crosstalk of blue light on Vf-Chrimson. (D) Photocurrents for the first 50 neurons.

**Extended Data Fig. 4:**
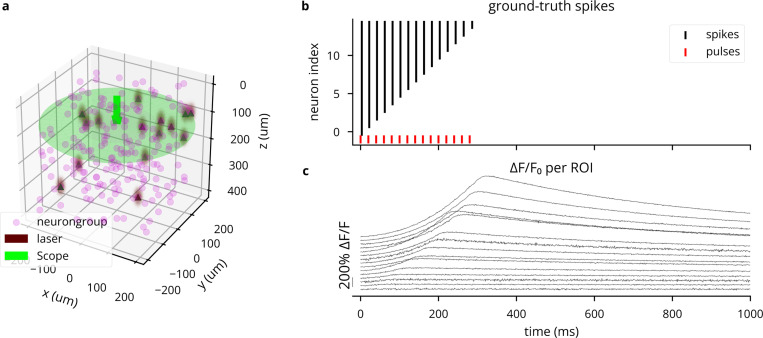
Simulation of two-photon calcium imaging using the GCaMP6f indicator [86]. (A) 3D plot of network model and microscope configuration. (B) Spike raster for the simulated experiment, where each ROI receives a number of laser pulses equal to its 1-based index. (C) $\text{Δ}F/F_{0}$ traces for each ROI, showing stronger responses for neurons having spiked more, but varying with expression levels. Heterogeneity in noise is due to varying distances from the focal plane.

**Extended Data Fig. 5:**
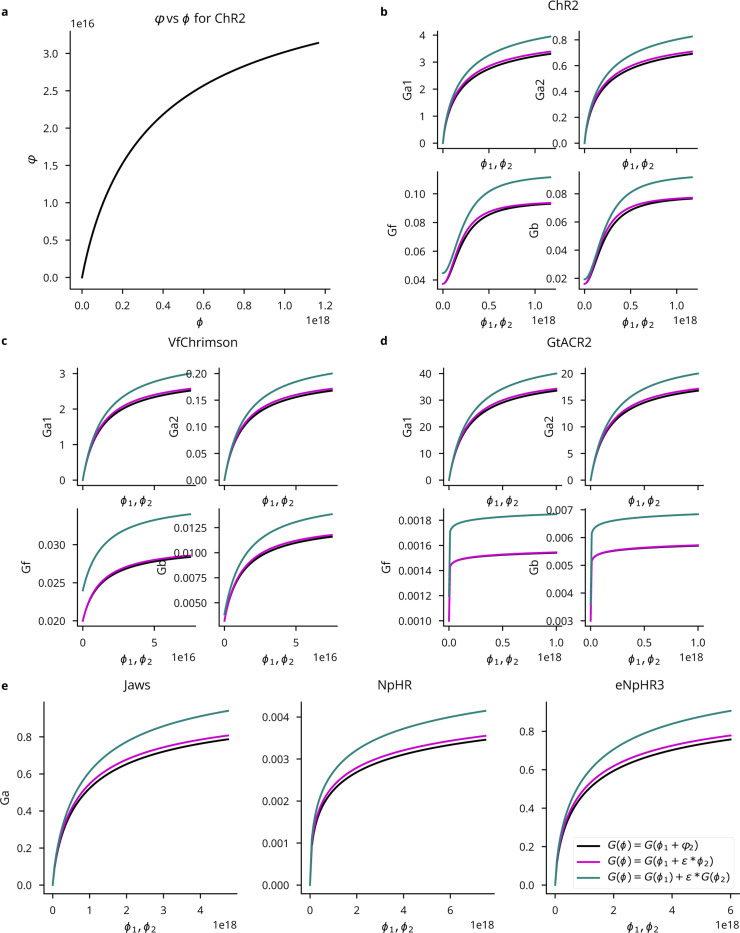
Multi-wavelength opsin model comparison. $\phi_{1}$, $\phi_{2}$ refer to photon flux at peak wavelength ${\text{λ}}_{1}$ and some other wavelength ${\text{λ}}_{2}$, respectively. All panels take ε=0.2 and use the legend in *E*. (A) The computed effective flux φ at ${\text{λ}}_{2}$ as a function of the actual flux ϕ. (B-D) Light-dependent activation functions for four-state ChR2, Vf-Chrimson, and GtACR2 opsins. (E) Light-dependent activation for the three-state anion pump models. Parameters given in [59].

**Extended Data Fig. 6:**
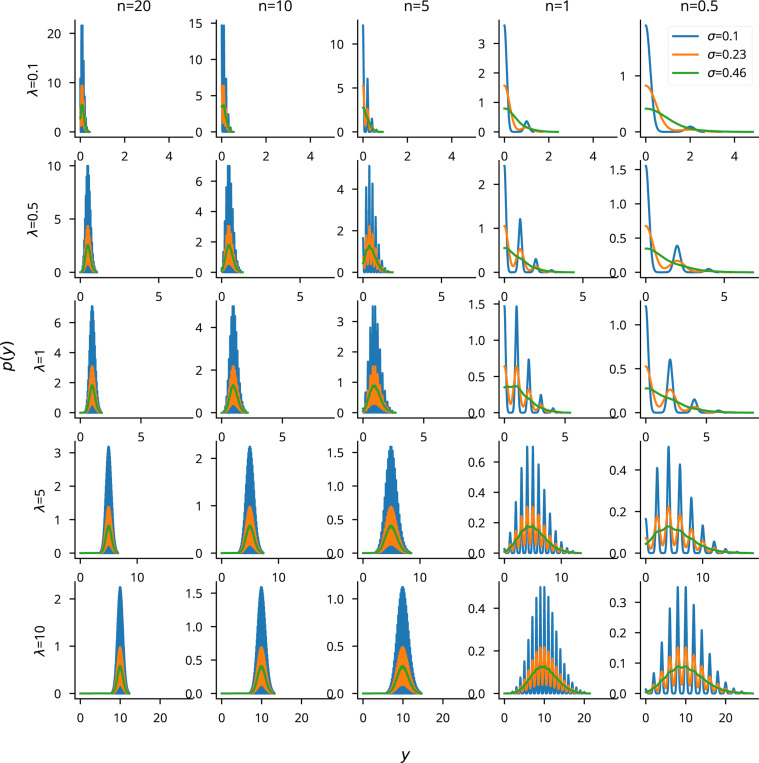
A visualization to assess the appropriateness of the Gaussian noise model for imaging experiments. We plot the Gaussian distribution p(y)=𝓝(x,σ) over a Poisson photon count per pixel x∼Pois(λ). N refers to the number of pixels visible in the ROI and λ is the expected photon count. Plots show a roughly Gaussian-distributed p(y) when N>1, which is a realistic assumption for imaging experiments. The spikiness would be mitigated in a real experiment, where λ and σ would not be constant across pixels. The Gaussian observation appears to be least appropriate for low photon counts, where the distribution has a heavy right tail.

**Extended Data Fig. 7:**
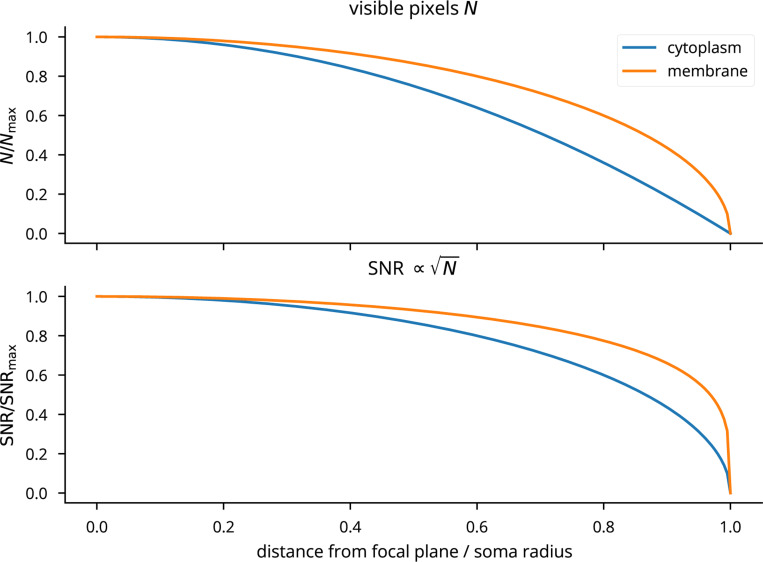
Plot of the number of visible pixels N and the SNR as a function of the distance from the focal plane, for indicators found both in the cytoplasm (calcium indicators) and membrane (voltage indicators).

**Extended Data Fig. 8:**
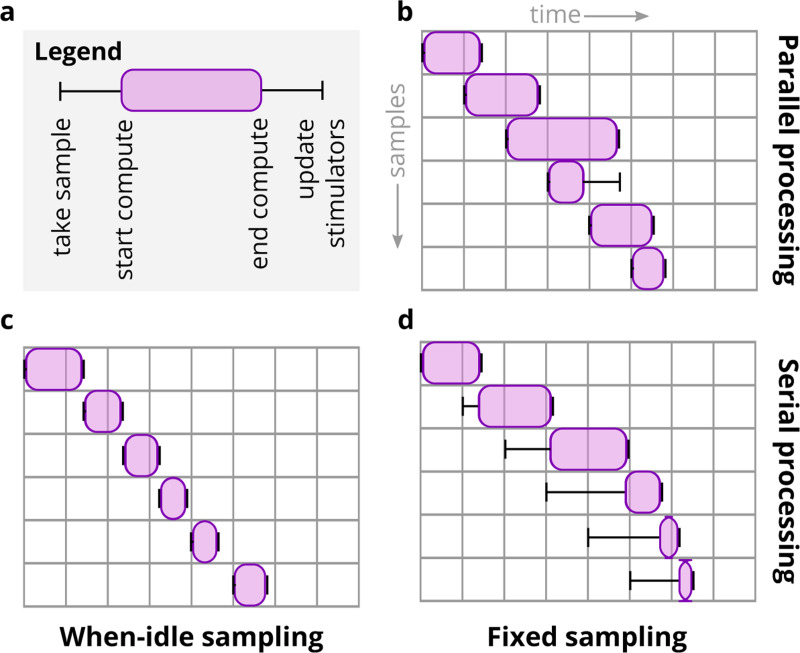
Latency emulation strategy and available configurations. (A) Cleo registers the time a sample is taken from the recording devices, determines the times the computation starts and ends, applies the user-specified delay, and updates stimulation devices when finished. (B) The default parallel processing/fixed sampling mode. Updates are reserved until the previous update is delivered so the sequence of stimulator updates corresponds to the sequence of measurements. (C) The “when-idle” processing mode samples only once the computation for the previous step has terminated. (D) The serial processing/fixed sampling case reflects when computations are not performed in parallel, but sampling continues on a fixed schedule. Samples are taken either as soon as possible after the previous sample time was missed, or on schedule otherwise.

**Extended Data Fig. 9:**
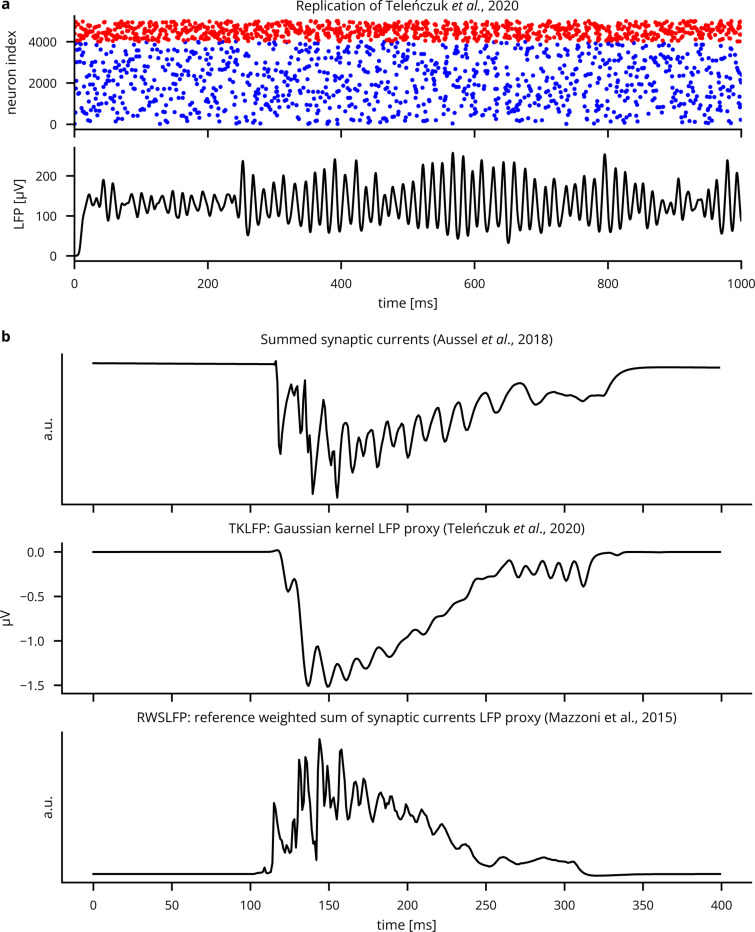
Validation of LFP proxy methods. (A) Replication of the Teleńczuk kernel LFP demo [76]. (B) Comparison of LFP proxy signals during SWR-like activity in a hippocampus model (see Sec. 3.3.3). Aussel *et al*. represent LFP with a sum of synaptic currents, each neuron’s contribution depending on its location in space [98]. The Gaussian kernel approximation method is as described in [76] and computed by Cleo, which uses the tklfp package implementation [80]. The reference weighted sum method is described in [77] and is also computed by Cleo, which uses the wslfp package implementation [81] (see Sec. 2.4.2).

**Extended Data Fig. 10:**
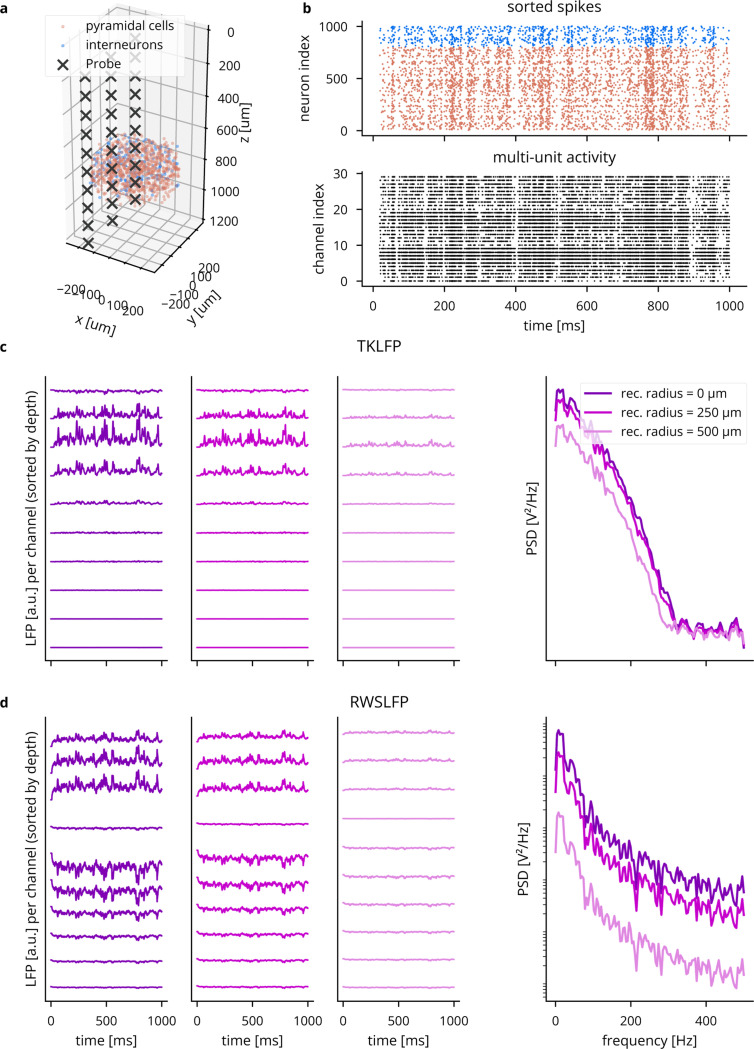
Comparison of the TKLFP and RWSLFP proxy methods for a simulated E/I network. We see here that TKLFP captures less high-frequency content, which is as reported by Teleńczuk *et al*. (A) A Cleo-generated plot of the network model and electrode placement. (B) Sorted and multi-unit spiking activity recorded from the network. (C) LFP and power spectral density (PSD) for the TKLFP signal recorded by the electrode. (D) Same as C, but for the RWSLFP signal.

## Supplementary Material

Supplement 1

## Figures and Tables

**Table 1:** Feature comparison of experiment simulation software.

Software	Simulator	Ideal for point neurons	Closed-loop stimulation	Opsin kinetics models	Reconfigurable illumination	Extracellular recording	2P imaging
Mozaik [31]	Multiple (through PyNN)	✓	✓	✓	1P	†	
LFPy 2.0 [42]	NEURON			✓		✓	
BioNet [34, 35]	NEURON		✓	✓		✓	
NetPyNE [36]	NEURON			✓		✓	
VERTEX 2.0 [32, 33]	Custom MATLAB		✓ − (inflexible)			✓	
NAOMi [44]	Custom MATLAB				2P		✓
Cleo	Brian 2	✓	✓ + (w / latency)	✓	1P & 2P	✓ − (approx.)	✓

†: Mozaik can record spikes from a subset of neurons selected by proximity to electrodes, but does not simulate LFP or spike detection noise as a function of distance from the electrode.

**Table 2:** Experiment computation details. Runtimes describe individual conditions/trials, rather than the entire experiment.

Experiment	Computer	Cleo version	Sim. time	Approximate runtime
VE1: HPC seizure recording	Dell laptop	v0.14.1	35 s	210 min
VE2: All-optical control	Dell laptop	v0.15.0	800 ms	15/1.5 s with/without imaging
VE3: Bidirectional optoclamp	Dell laptop	v0.15.0	90s	30 min
PE1: Traveling wave rejection	Dell laptop	v0.15.0	15 ms	30 s, including setup
PE2: V1 plasticity disruption	PACE	v0.8.0	137 s	60/45 min with/without Cleo
PE3: SWR evocation	Dell laptop	v0.10.0	400 ms	5/4 min with/without opto

VE: validation experiment, PE: prospective experiment.
