# Supplementary material for: Bridging model and experiment in systems neuroscience with Cleo: the Closed-Loop, Electrophysiology, and Optophysiology simulation testbed
Source: bioRxiv. 2025 Aug 23:2023.01.27.525963. Preprint. [Version 3] doi: 10.1101/2023.01.27.525963 (PMC11257437; doi:10.1101/2023.01.27.525963)
Supplement: Supplement 1 [file NIHPP2023.01.27.525963v3-supplement-1.pdf]

bioRxiv preprint doi: https://doi.org/10.1101/2023.01.27.525963; this version posted August 23, 2025. The copyright holder for this preprint (which was not certified by peer review) is the author/funder, who has granted bioRxiv a license to display the preprint in perpetuity. It is made available under aCC-BY 4.0 International license.

# 7. Supplementary Methods

## 7.1. Multi-wavelength sensitivity

### 7.1.1. Light superposition

Here we describe how we model dynamics for light of two different wavelengths, given photon flux $\phi_{\lambda_1}$ and $\phi_{\lambda_2}$. Bansal et al. (2020b) use a weighted sum of activation functions

$$G(\phi) = G(\phi_{\lambda_1}) + \varepsilon G(\phi_{\lambda_2}).$$

However, these are sublinear functions, so adding them results in exaggerated activation. In the extreme case, imagine two light sources that are just 1 nm apart in wavelength, with $\varepsilon = 1$. Thus:

$$\phi_{\lambda_1} = \phi_{\lambda_2} = \phi_{tot}/2$$
$$G(\phi_{tot}) \approx G(\phi_{\lambda_1}) + G(\phi_{\lambda_2}) = 2G(\phi_{tot}/2),$$

which contradicts what we expect for sublinear $G$:

$$G(\phi) < 2G(\phi/2)$$

A more accurate approach instead assumes the following

$$G(\varphi_{\lambda_2}) = \varepsilon G(\phi_{\lambda_2}),$$

where $\varphi_{\lambda_2}$ is the standard-wavelength equivalent ("effective flux") of $\phi_{\lambda_2}$.

Demonstrating with $G_{a1}$ of the four-state model (Evans et al., 2016), we solve for $\varphi$ in terms of $\phi$ and $\varepsilon$:

$$\begin{aligned}
G_{a1}(\varphi) &= \varepsilon G_{a1}(\phi) \\
\frac{\varphi^p}{\varphi^p + \phi_m^p} &= \varepsilon \frac{\phi^p}{\phi^p + \phi_m^p} \\
\varphi^p \phi^p + \varphi^p \phi_m^p &= \varepsilon(\varphi^p + \phi_m^p)\phi^p \\
\left(\phi^p + \phi_m^p - \varepsilon\phi^p\right)\varphi^p &= \varepsilon\phi^p\phi_m^p \\
\varphi^p &= \frac{\varepsilon\phi^p\phi_m^p}{(1-\varepsilon)\phi^p + \phi_m^p} \\
\varphi &= \left(\frac{\varepsilon\phi^p\phi_m^p}{(1-\varepsilon)\phi^p + \phi_m^p}\right)^{1/p}.
\end{aligned}$$

bioRxiv preprint doi: https://doi.org/10.1101/2023.01.27.525963; this version posted August 23, 2025. The copyright holder for this preprint (which was not certified by peer review) is the author/funder, who has granted bioRxiv a license to display the preprint in perpetuity. It is made available under aCC-BY 4.0 International license.

We then compute our activation functions as

$$G(\phi_{\lambda_1}, \phi_{\lambda_2}) = G(\phi_{\lambda_1} + \varphi_{\lambda_2}).$$

Unfortunately, this doesn't yield a simple constant conversion factor. However, if we approximate $G$ as linear, we can use a weighted sum of fluxes:

$$G(\phi) = G(\phi_{\lambda_1} + \varepsilon\phi_{\lambda_2})$$

Plotting $G$ for multiple opsins shows this linear approximation does yield a lower activation curve than the Bansal *et al.* approach and is qualitatively close to the true $\varphi$ derived above. See Fig. S6 for a comparison of the three methods.

#### *7.1.2. Action spectrum normalization*

Some action spectra are measured with equal power density/pulse width across wavelengths, while others are reported with equal photon flux. We store ours as equal power density spectra since they seem to be more common and allow for the opsin model to use both accurate power density and photon flux values. We use $\varepsilon$ to represent sensitivity relative to the peak-sensitivity wavelength, $\varepsilon_\phi$ and $\varepsilon_P$ representing the equal photon flux and power density versions, respectively.

For a given wavelength $\lambda$,

$$\varepsilon(\lambda) = \frac{G(\varphi_\lambda)}{G(\phi)} = \frac{G_\lambda}{G(\phi)}.$$

We let $G_\lambda$ represent the response at wavelength $\lambda$, while $G(\phi)$ represents the response at the peak wavelength for the same flux $\phi$. We will assume $G$ is a linear function, as above, using $C$ to represent a constant:

$$\varepsilon_\phi(\lambda) = \frac{G_\lambda}{G(\phi)} \qquad \varepsilon_P(\lambda) = \frac{G_\lambda}{G(\phi)}$$
$$\varepsilon_\phi(\lambda) = \frac{G_\lambda}{C\phi} \qquad \varepsilon_P(\lambda) = \frac{G_\lambda}{C\phi}$$

bioRxiv preprint doi: https://doi.org/10.1101/2023.01.27.525963; this version posted August 23, 2025. The copyright holder for this preprint (which was not certified by peer review) is the author/funder, who has granted bioRxiv a license to display the preprint in perpetuity. It is made available under aCC-BY 4.0 International license.

Then we make either photon flux or irradiance (power density) constant:

$$\varepsilon_\phi(\lambda) = \frac{G_\lambda}{C\phi_{\text{const}}} \qquad \varepsilon_P(\lambda) = \frac{G_\lambda}{CI_{\text{const}}/e_{\text{photon}}}$$
$$\varepsilon_P(\lambda) = \frac{G_\lambda}{CI_{\text{const}}\lambda}$$
$$\varepsilon_\phi(\lambda) = \frac{G_\lambda}{C} \qquad \varepsilon_P(\lambda) = \frac{G_\lambda}{C\lambda}.$$

$$C\varepsilon_\phi(\lambda) = C\lambda\varepsilon_P(\lambda) = G_\lambda$$

$$\varepsilon_P(\lambda) = C\frac{\varepsilon_\phi(\lambda)}{\lambda}$$

Thus, we can convert from $\varepsilon_\phi(\lambda)$ to $\varepsilon_P(\lambda)$ by dividing by $\lambda$ and normalizing.

### *7.2. GECI convolution simulation as an ODE*

Song *et al.* convolve the intracellular calcium trace $\left[\text{Ca}^{2+}\right]$ with a double exponential kernel to capture variable rise and decay times in the fluorescence signal. To simplify simulation (to not have to keep a buffer of past calcium values), we can represent this convolution as an ODE. Let $c(t)$ and $b(t)$ be the free and bound calcium concentrations and $h(t)$ be the kernel function

$$h(t) = u(t)A\left(1 - e^{-t/\tau_{\text{on}}}\right)e^{-t/\tau_{\text{off}}}$$

$$b(t) = c(t) * h(t),$$

where $u(t)$ is the unit step function, included to ensure the kernel is causal.

We can represent the convolution as multiplication in the Laplace domain:

$$B(s) = C(s)H(s).$$

By expanding out $h(t)$, we get functions that can easily be transformed into the Laplace domain. Let $\kappa = \tau_{\text{off}}^{-1}, \lambda = \tau_{\text{off}}^{-1} + \tau_{\text{on}}^{-1}$ to simplify notation.

bioRxiv preprint doi: https://doi.org/10.1101/2023.01.27.525963; this version posted August 23, 2025. The copyright holder for this preprint (which was not certified by peer review) is the author/funder, who has granted bioRxiv a license to display the preprint in perpetuity. It is made available under aCC-BY 4.0 International license.

$$\begin{aligned}
h(t) &= Au(t)\left(1 - e^{-T_{\mathrm{on}} t}\right) e^{-T_{\mathrm{off}} t} \\
&= A(u(t)e^{-\kappa t} - u(t)e^{-\lambda t}) \\
H(s) &= A\left(\frac{1}{s+\kappa} - \frac{1}{s+\lambda}\right) \\
B(s) &= A\left(\frac{1}{s+\kappa} - \frac{1}{s+\lambda}\right) C(s).
\end{aligned}$$

Now we get a common denominator and rearrange:

$$\begin{aligned}
B(s) &= A\left(\frac{\lambda-\kappa}{(s+\kappa)(s+\lambda)}\right) C(s) \\
B(s) &= A\left(\frac{\lambda-\kappa}{s^2+(\kappa+\lambda)s+\kappa\lambda}\right) C(s) \\
\left(s^2+(\kappa+\lambda)s+\kappa\lambda\right) B(s) &= A\left(\lambda-\kappa\right) C(s)
\end{aligned}$$

Now we use the $s^2$ and $s$ terms to convert to a second-order ODE, using the fact that the Laplace transform of $b''(t)$ and $b'(t)$ are $s^2B(s) - sb(0^-) - b(0^-)$ and $sB(s) - b(0^-)$, respectively. Also, we assume that $b(0) = b'(0) = 0$ to avoid undefined $\delta(t)$ and $\delta'(t)$ terms after the inverse Laplace transform; this appears to have only a minor effect.

$$b''(t) + (\kappa+\lambda)b'(t) + \kappa\lambda b(t) = A(\lambda-\kappa)c(t).$$

Rearranging to a first-order ODE system by introducing $\beta(t) = b'(t)$, we get

$$\begin{aligned}
b'(t) &= \beta(t) \\
b''(t) &= \beta'(t) = A(\lambda-\kappa)c(t) - (\kappa+\lambda)\beta(t) - \kappa\lambda b(t).
\end{aligned}$$

Special thanks to DinosaurEgg on Math Stack Exchange for helping solve this problem.

### *7.3. Probabilistic spike detection details*

To approximate multi-unit and sorted spike recording without simulating full extracellular action potential (EAP) waveforms, Cleo takes ground-truth spikes returned by the Brian simulator, makes noisy measurements of their amplitudes alone, and stochastically determines which to report as recorded on the probe. The recorded amplitude of an average spike $A_{\mu s}$ is a function of $r$, the distance between the neuron and the electrode (see Fig. 3a). This function is parameterized by $r_{\text{noise floor}}$, the distance at which signal-to-noise ratio (SNR) is 1, i.e., $A_{\mu s}(r_{\text{noise floor}}) \triangleq \sigma_b \triangleq 1$, where $\sigma_b$ is the standard deviation of background (electrical

bioRxiv preprint doi: https://doi.org/10.1101/2023.01.27.525963; this version posted August 23, 2025. The copyright holder for this preprint (which was not certified by peer review) is the author/funder, who has granted bioRxiv a license to display the preprint in perpetuity. It is made available under aCC-BY 4.0 International license.

and biological) noise. The default $r_{\text{noise floor}}$ is 80 µm, as reported by Cohen and Miles (Cohen and Miles, 2000), which is consistent with other literature stating the measured amplitude drops to near 0 between 140 and 300 µm (Henze et al., 2000; Somogyvári et al., 2005). Cleaner/noisier recordings can be achieved by lengthening/shortening $r_{\text{noise floor}}$.

The shape of the amplitude profile is defined by $\widetilde{A}_{\mu s}(r)$, which can take any monotonically decreasing form, but is $1/r^2$ by default. This follows detailed simulations by Pettersen and Einevoll (Pettersen and Einevoll, 2008) that show $A_{\mu s}$decreasing with $1/r^n$, where $n$ varies between 2 and 3 depending on cell type and distance. The raw $\widetilde{A}_{\mu s}(r)$ function is shifted by a small distance $r_0 = 5$ µm to avoid division by 0 and scaled such that $A_{\mu s}(r_{\text{noise floor}}) = \sigma_b$:

$$A_{\mu s}(r) = \frac{\widetilde{A}_{\mu s}(r + r_0)}{\widetilde{A}_{\mu s}(r_{\text{noise floor}} + r_0)}. \tag{12}$$

Variability in measured EAP amplitudes comes from the aforementioned background noise as well as variability in spike amplitude. The former is simulated as white noise in the spiking band, independent across channels, and scaled to have standard deviation $\sigma_b$. To ensure the noise has consistent energy and spectral properties with different temporal resolutions, we produce a spiking band-limited noise waveform per channel. An alternative of a simple Gaussian sample every time step, for example, would produce more false positives the smaller the time step. Cleo produces this noise by filtering per-time step white noise with a 6th-order Butterworth filter with cutoff frequencies of 300 and $\min(3000, 0.45 f_s)$ Hz, the upper cutoff being adjusted to stay under the Nyquist frequency.

Intrinsic amplitude variability is modeled as a Gaussian distribution around the mean with standard deviation $\text{CV}_A A_{\mu s}$. $\text{CV}_A$ signifies the coefficient of variation and is 0.05 by default. This default was estimated from the median of 20 randomly selected reporter-positive mouse cells from the Allen Cell Types Database (all). For each cell, we selected the sweep with the most spikes and computed the coefficient of variation of spike amplitudes, where amplitudes were taken to be the difference between the peak and threshold voltages for each spike.

Combining variation from these two sources, the variance in measured amplitude across spikes is thus $\sigma_A^2(r) = (A_{\mu s}(r)\text{CV}_A)^2 + \sigma_b^2$. With $A_{\mu s}(r)$, $\sigma_A(r)$, and detection threshold $\vartheta$, we can compute the probability of detecting a given spike from a neuron on a single channel at distance $r$ as

$$p_{\text{detect}}(r) = 1 - \Phi\left(\frac{\vartheta - A_{\mu s}(r)}{\sigma_A(r)}\right), \tag{13}$$

bioRxiv preprint doi: https://doi.org/10.1101/2023.01.27.525963; this version posted August 23, 2025. The copyright holder for this preprint (which was not certified by peer review) is the author/funder, who has granted bioRxiv a license to display the preprint in perpetuity. It is made available under aCC-BY 4.0 International license.

where $\Phi$ is the CDF of the standard normal distribution (see Fig. 3a).

In large simulations, it would be computationally expensive to sample threshold crossings and especially collisions for neurons that are distant from the electrode and/or each other. Thus, when a probe is injected into a neuron group, Cleo computes the multi-channel recall for each neuron as

$$p_{\text{detect,multi}}(\{r_c\}) = 1 - \left(\prod_c 1 - p_{\text{detect}}(r_c)\right), \tag{14}$$

where $r_c$ is the distance from the neuron to channel $c$. The probe then subscribes only to neurons with $p_{\text{detect,multi}} > 0.001$, thus including fairly low-SNR neurons but ruling out the very distant. Note that while noise is independent across channels, the intrinsic spike amplitude is shared. The independence assumption in Equation 14 is thus an approximation that saves computational effort by removing the correlation cross-terms across channels.

Candidate threshold crossings are identified from spikes and non-spike noise values that exceed $\vartheta$, by default $4\sigma_b$. To efficiently consider all spikes on a given channel in parallel and support varying temporal resolution, they are assessed independently despite the fact that in a real experiment there is only one measurement on a channel at a given time. I.e., in our method two subthreshold measurements do not combine to produce a threshold crossing. While this approach may seem unrealistic, it avoids another unrealistic scenario where many distant spikes combine to regularly exceed the threshold. This also supports varying temporal resolution in the sense that it allows us to use coarser temporal resolution without limiting how many "measurements" can be made at each time step per channel.

These threshold crossings then go through a “collision” sampling process modeling limitations in detecting overlapping spikes. This is parameterized by a collision probability function $p_{\text{coll}}(\Delta t)$, where $\Delta t$ is the time difference between candidate threshold crossings. To capture the common practice of setting a minimum refractory period in MUA, the default for multi-unit activity is $p_{\text{coll,MUA}} = \mathbf{1}_{\{\Delta t < 1\,\text{ms}\}}$, meaning that collision is certain for spikes within 1 ms of each other. Reflecting the behavior typical of many spike sorters (Garcia et al., 2022), the default for sorted spiking is an exponential decay function $p_{\text{coll,SS}} = 0.2\exp(-\Delta t/0.3\,\text{ms})$ (see Fig. 3b). Every spike is compared to previous spikes in the recent past and in the case of collision, the first one is kept (to preserve the causal nature of the simulation). For simultaneous spikes, the one with the highest amplitude is kept.

The resulting output is then processed to produce the final multi-unit or sorted output (for an example, see Fig. 3c,d). Multi-unit activity reports every spike detected by every channel, without regard for the origin

bioRxiv preprint doi: https://doi.org/10.1101/2023.01.27.525963; this version posted August 23, 2025. The copyright holder for this preprint (which was not certified by peer review) is the author/funder, who has granted bioRxiv a license to display the preprint in perpetuity. It is made available under aCC-BY 4.0 International license.

of the spike (though with the default collision function, there will never be more than one per time step per channel). Sorted spiking, on the other hand, reports all spikes detected on at least one channel, where each neuron is identified by a unique index. Only neurons with a SNR (defined as the peak $A_{\mu s}/\sigma_A$ across channels) above a threshold (default $6\sigma_b$) are included in the sorted output, reminiscent of SNR filtering of spikes in some sorters (Yger et al., 2018) and analyses (Magland et al., 2020). While real-time spike sorting for closed-loop control is difficult in practice for large channel counts, this sorted spiking option could be used to emulate a more common workflow of isolating one or a few neurons to record spikes from in real time. Note that we simplify the sorting process in several ways: (1) by assuming perfect sorting, (2) by treating the detection process as an independent process per channel, not incorporating spatial patterns as spike sorters do, (3) by keeping the first spike in case of collisions, rather than throwing out both or misclassifying one, and (4) reporting no false positives, since threshold crossings from noise alone would very rarely match a template. As a consequence of the last point, the downside to lowering the detection threshold for sorted spikes is that false positives can increase spikes missed due to collisions.

### *7.4. Bayesian optimization of neuron models for light pulse characterizations*

Neuron parameters were selected for AdEx and LIF neurons to match the firing rates of the source simulations in (Foutz et al., 2012) via Bayesian optimization. We used the Bayesian Optimization Python package (Nogueira, 2014 ; Gardner et al., 2014), primed with four parameter settings from the Neuronal Dynamics Textbook (Gerstner et al., 2014) and 20 random settings, followed by 200 iterations of optimization. The objective function was the sum of squared error between the target and simulated firing rates, totalled across all five conditions, i.e., 120%/75%, 120%/100%, 120%/150%, 100%/100%, and 140%/100% light intensity/expression level combinations. 100% light intensity was defined as that required to produce a single spike with a 5 ms pulse.

### *7.5. Prospective experiment 3*

The reference signal was generated by delivering a 1 nA square wave input from 100 to 300 ms to entorhinal cortex using the original model's $I_{\text{ext}}$ term, without noise added. Training data was generated by running the system for 13 seconds with alternating on and off periods of length $T \sim \mathcal{N}(200,\ 50)$ µs. During "on" intervals, $I_{\text{ext}} \sim |\mathcal{N}(0, \text{Irr}_{\max}/3)|$. $\text{Irr}_{\max}$ was 75 mW/mm$^2$, which is described as an upper safety limit for 473 nm light delivery to the brain (Cardin et al., 2010). Gaussian process noise was generated with mean

bioRxiv preprint doi: https://doi.org/10.1101/2023.01.27.525963; this version posted August 23, 2025. The copyright holder for this preprint (which was not certified by peer review) is the author/funder, who has granted bioRxiv a license to display the preprint in perpetuity. It is made available under aCC-BY 4.0 International license.

$\mu = 0.167\,\text{nA}$ and using the exponentiated quadratic kernel

$$k(t_1, t_2) = \sigma^2 e^{\frac{-(t_2-t_1)^2}{2l^2}} \tag{15}$$

with $\sigma = 0.083\,\text{nA}, l = 30\,\text{ms}$ and was added to input current $I_{\text{ext}}$ in each control scenario. The parameters of light delivery were altered from the 473 nm-wavelength optic fiber defaults to allow for greater propagation—$K$ and $S$ were both divided by 10. The training data, sampled at 1 kHz, was fit using the ldsCtrlEst library's SSID and EM fitting methods with latent dimensionality $n_x = 4$ (Bolus et al., 2022). An SSID fit was performed first, later refined by EM. ldsCtrlEst's Gaussian linear quadratic regulator (LQR) controller was used with a gain computed from $Q = C^T C$, $R = 0.001$ state and input penalties, with a simulated 3 ms of latency.

Model-predictive control (MPC) was implemented with a control horizon of 50 time steps, i.e., 50 ms. The standard, quadratic cost function utilized the same $Q, R$ as LQR, and was optimized using the 'cvxpy' Python package (Diamond and Boyd, 2016). MPC was simulated with 6 ms latency.

## 8. Supplementary Figures

bioRxiv preprint doi: https://doi.org/10.1101/2023.01.27.525963; this version posted August 23, 2025. The copyright holder for this preprint (which was not certified by peer review) is the author/funder, who has granted bioRxiv a license to display the preprint in perpetuity. It is made available under aCC-BY 4.0 International license.

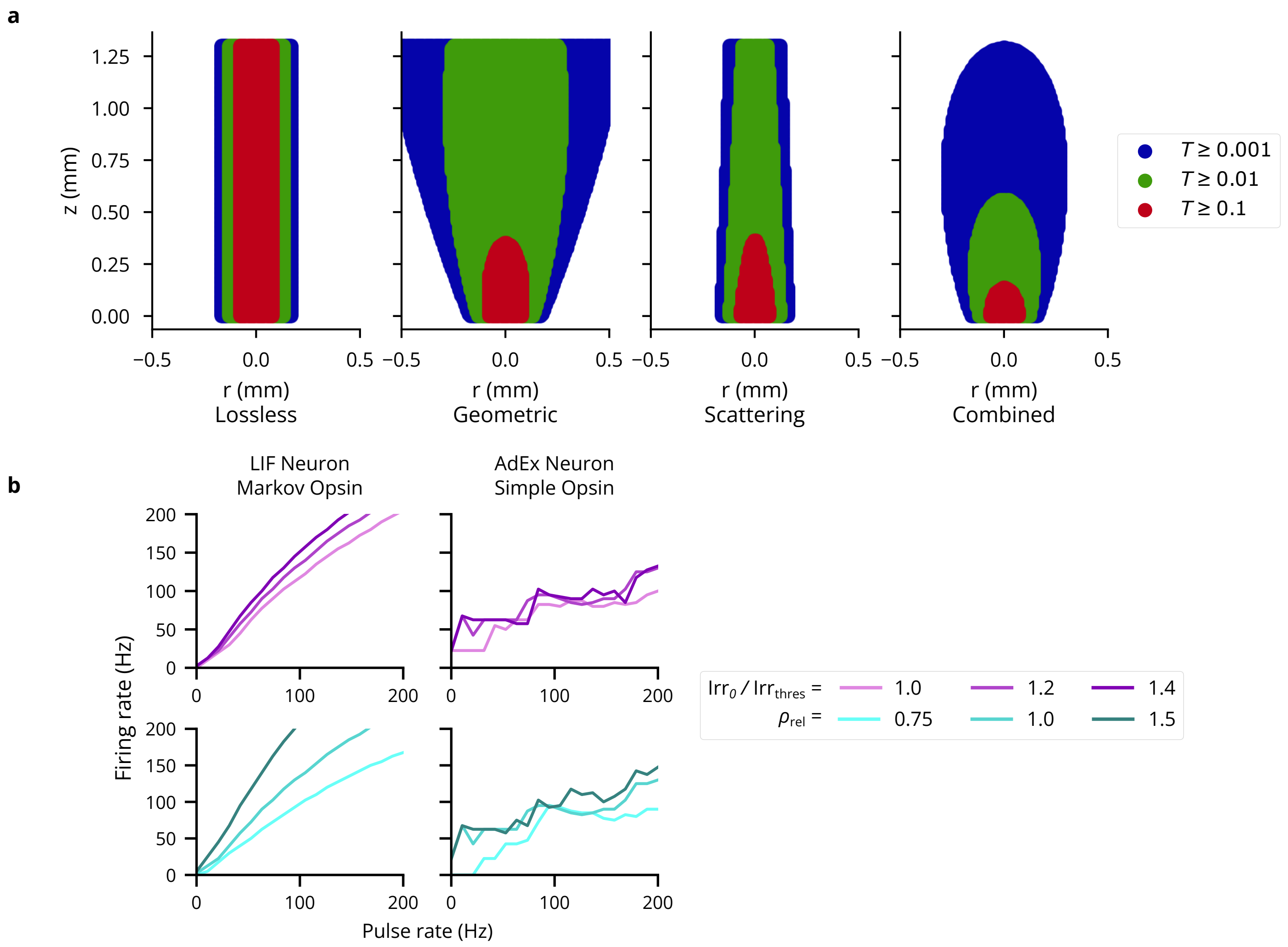


Figure S1: (A) Light transmittance $T$ as a function of radius and axial distance from the optic fiber tip (cf. Figure 2a from (Foutz et al., 2012)). The contribution of the Gaussian distribution, cone-shaped light propagation, and scattering are depicted separately. (B) Firing rate-pulse rate relationship as in Fig. 4c, for more neuron model-opsin combinations, namely LIF neuron with four-state Markov opsin and AdEx with a proportional current opsin. 5 ms pulses are used as before, with irradiance and expression levels as shown in the legend.

bioRxiv preprint doi: https://doi.org/10.1101/2023.01.27.525963; this version posted August 23, 2025. The copyright holder for this preprint (which was not certified by peer review) is the author/funder, who has granted bioRxiv a license to display the preprint in perpetuity. It is made available under aCC-BY 4.0 International license.

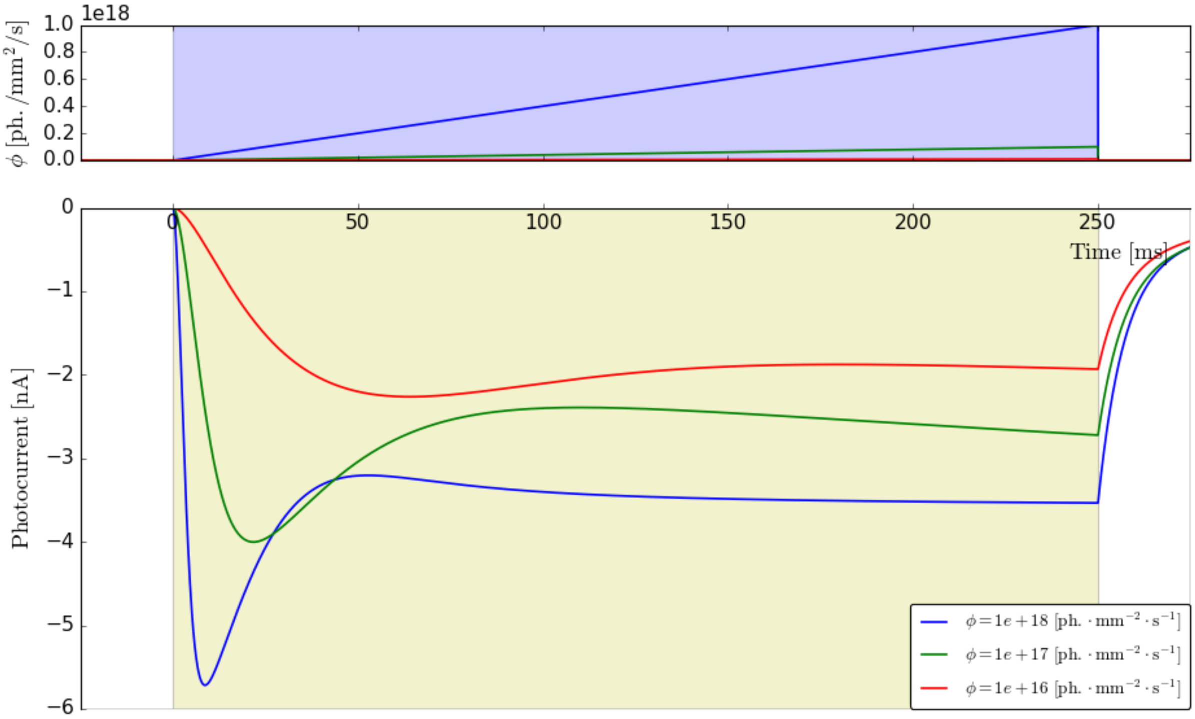


Figure S2: Output from the "ramp" protocol for the four-state ChR2 opsin model, produced by the PyRhO optogenetics simulation platform (Evans et al., 2016).

bioRxiv preprint doi: https://doi.org/10.1101/2023.01.27.525963; this version posted August 23, 2025. The copyright holder for this preprint (which was not certified by peer review) is the author/funder, who has granted bioRxiv a license to display the preprint in perpetuity. It is made available under aCC-BY 4.0 International license.

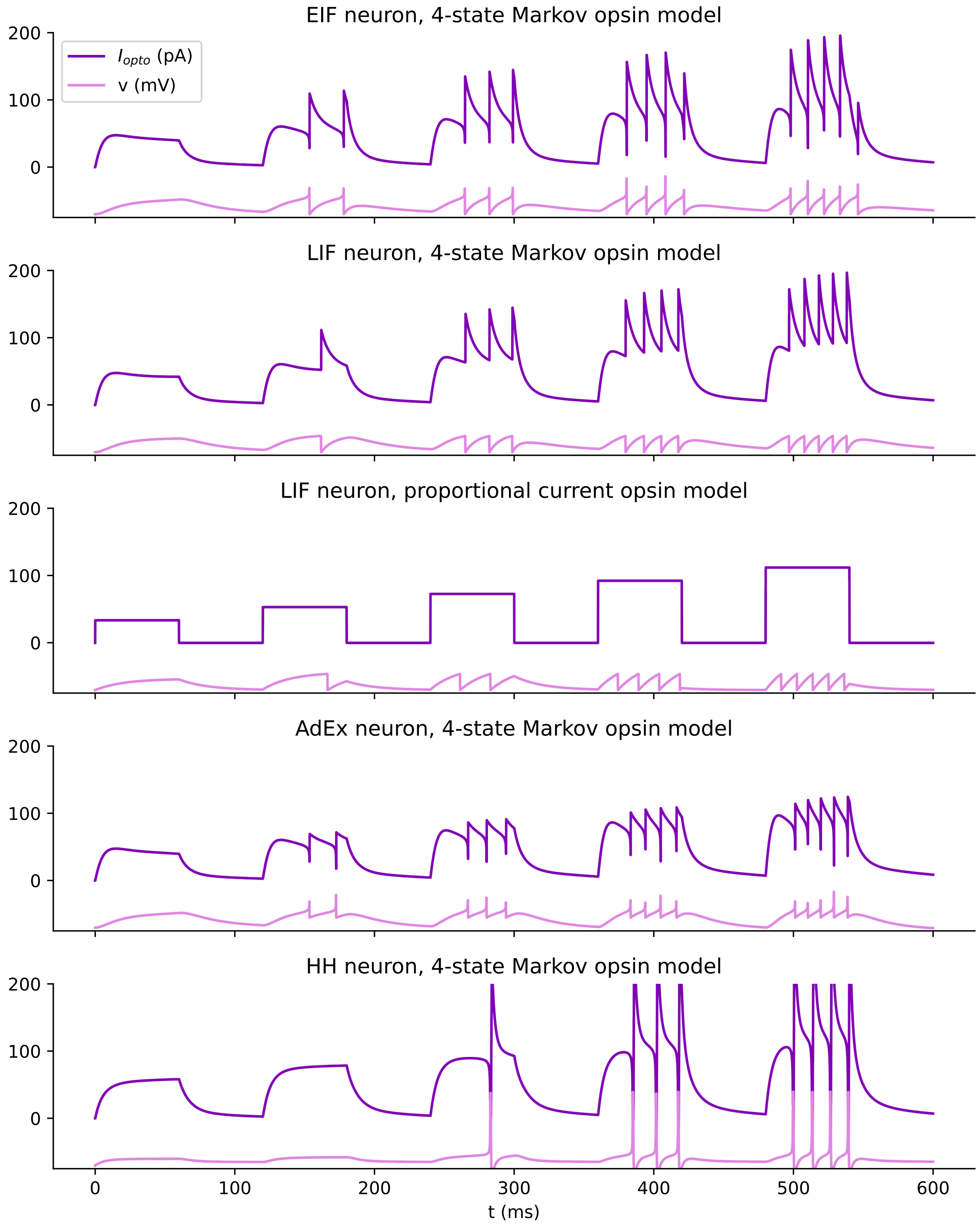


Figure S3: Responses of diverse opsin/neuron model combinations to light pulses of increasing intensity show qualitatively similar light-firing rate relationships.

bioRxiv preprint doi: https://doi.org/10.1101/2023.01.27.525963; this version posted August 23, 2025. The copyright holder for this preprint (which was not certified by peer review) is the author/funder, who has granted bioRxiv a license to display the preprint in perpetuity. It is made available under aCC-BY 4.0 International license.

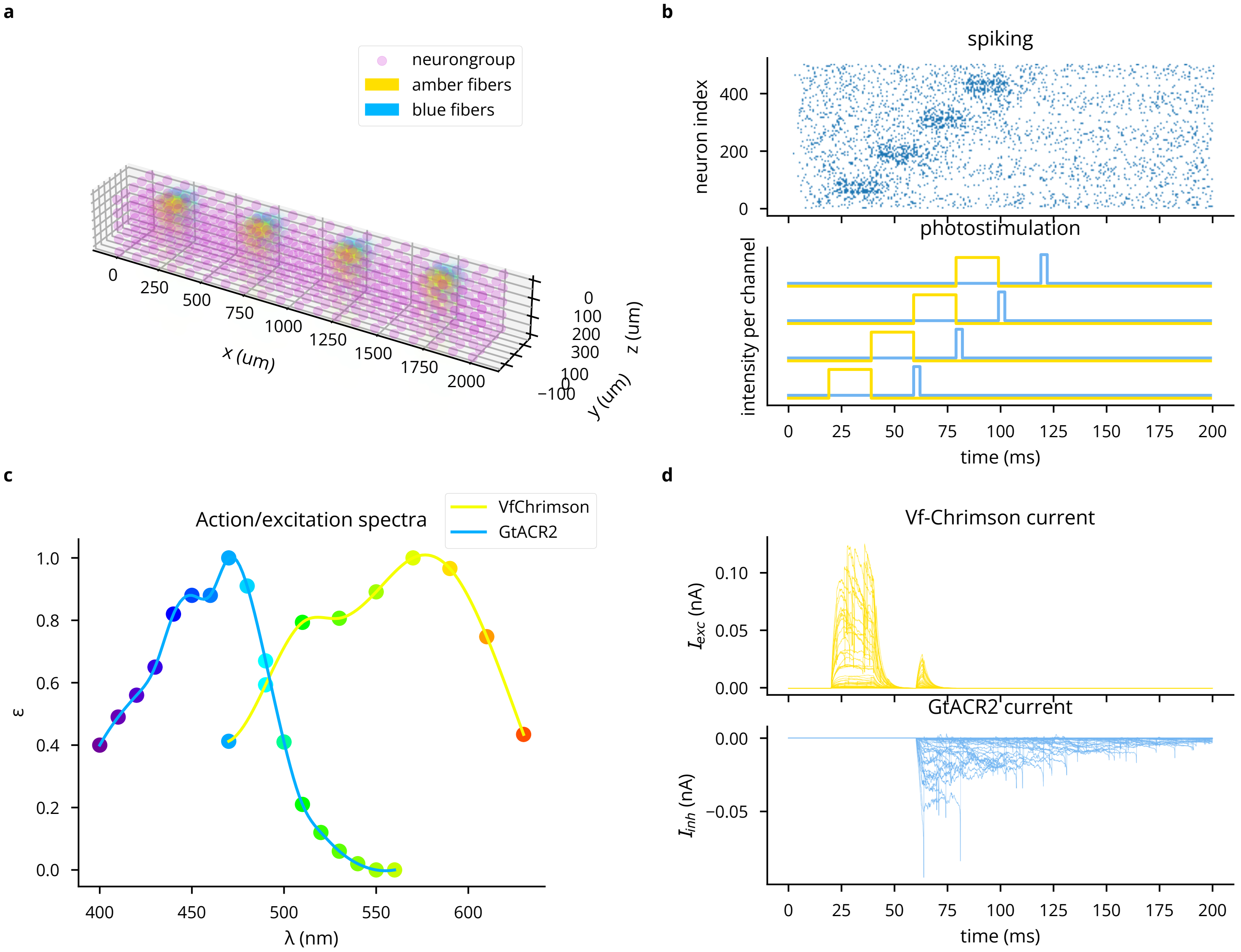


Figure S4: Demonstration of simulating multiple light sources, wavelengths, and opsins simultaneously. (A) 3D plot of network model and light sources. (B) Top: spike raster, where increasing neuron index correlates with increasing $x$ coordinates. Bottom: Stimulation pattern for 473 and 590 nm light sources. (C) Action spectra of Vf-Chrimson and GtACR2, showing crosstalk of blue light on Vf-Chrimson. (D) Photocurrents for the first 50 neurons.

bioRxiv preprint doi: https://doi.org/10.1101/2023.01.27.525963; this version posted August 23, 2025. The copyright holder for this preprint (which was not certified by peer review) is the author/funder, who has granted bioRxiv a license to display the preprint in perpetuity. It is made available under aCC-BY 4.0 International license.

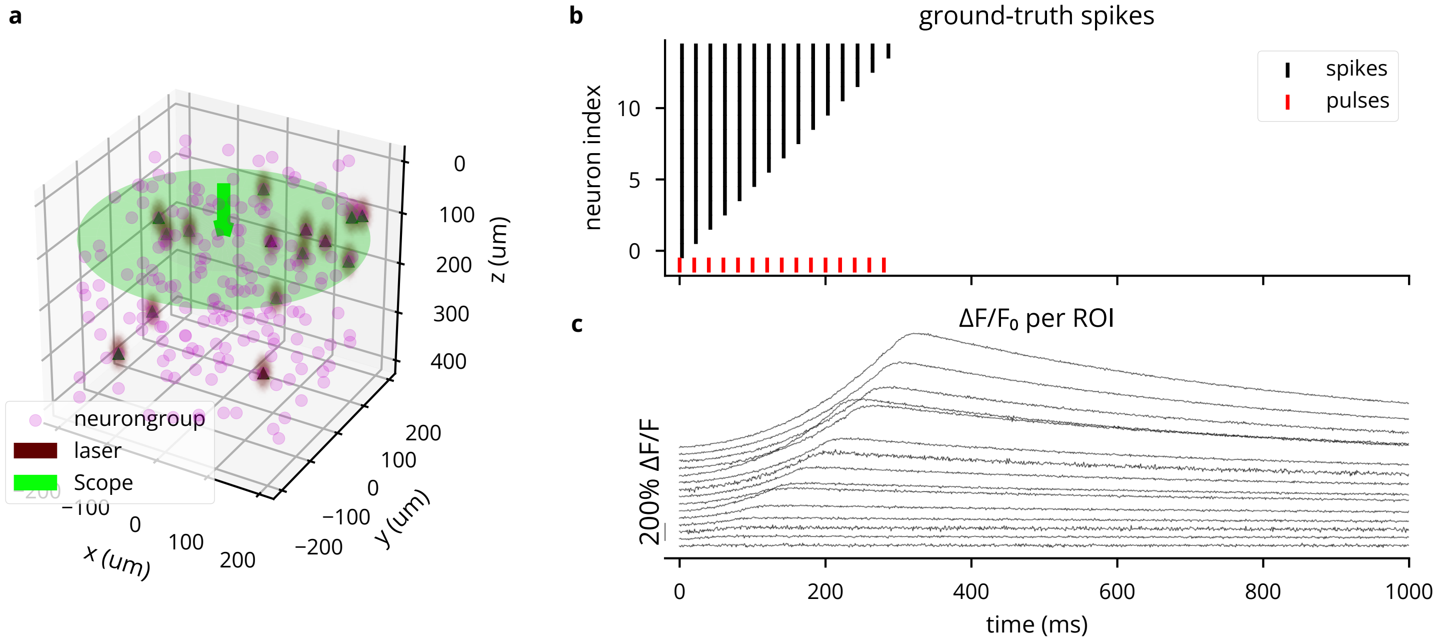


Figure S5: Simulation of two-photon calcium imaging using the GCaMP6f indicator (Badura et al., 2014). (A) 3D plot of network model and microscope configuration. (B) Spike raster for the simulated experiment, where each ROI receives a number of laser pulses equal to its 1-based index. (C) $\Delta F/F_0$ traces for each ROI, showing stronger responses for neurons having spiked more, but varying with expression levels. Heterogeneity in noise is due to varying distances from the focal plane.

bioRxiv preprint doi: https://doi.org/10.1101/2023.01.27.525963; this version posted August 23, 2025. The copyright holder for this preprint (which was not certified by peer review) is the author/funder, who has granted bioRxiv a license to display the preprint in perpetuity. It is made available under aCC-BY 4.0 International license.

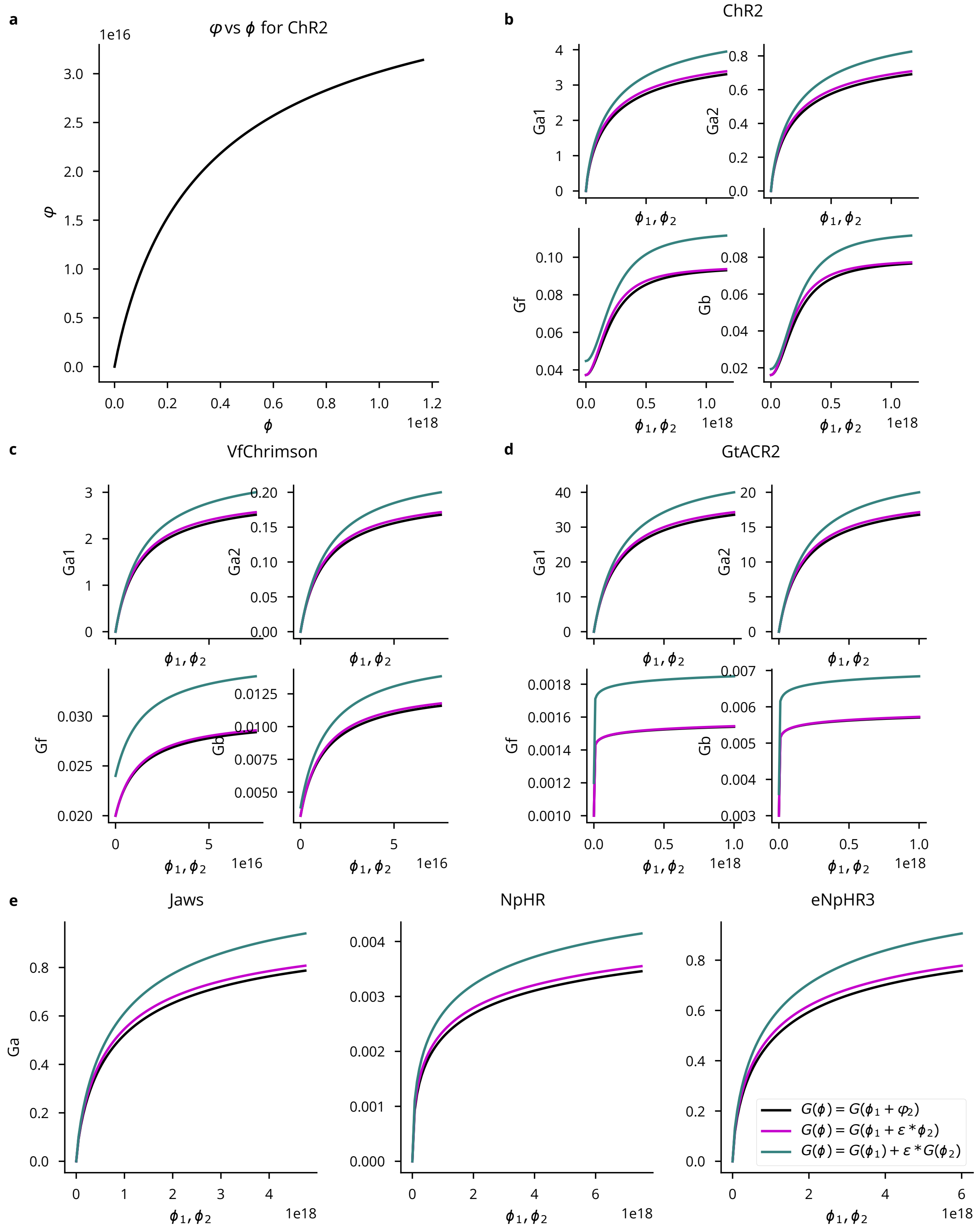


Figure S6: Multi-wavelength opsin model comparison. $\phi_1, \phi_2$ refer to photon flux at peak wavelength $\lambda_1$ and some other wavelength $\lambda_2$, respectively. All panels take $\varepsilon = 0.2$ and use the legend in *E*. (A) The computed effective flux $\varphi$ at $\lambda_2$ as a function of the actual flux $\phi$. (B-D) Light-dependent activation functions for four-state ChR2, Vf-Chrimson, and GtACR2 opsins. (E) Light-dependent activation for the three-state anion pump models. Parameters given in (Bansal et al., 2020b).

bioRxiv preprint doi: https://doi.org/10.1101/2023.01.27.525963; this version posted August 23, 2025. The copyright holder for this preprint (which was not certified by peer review) is the author/funder, who has granted bioRxiv a license to display the preprint in perpetuity. It is made available under aCC-BY 4.0 International license.

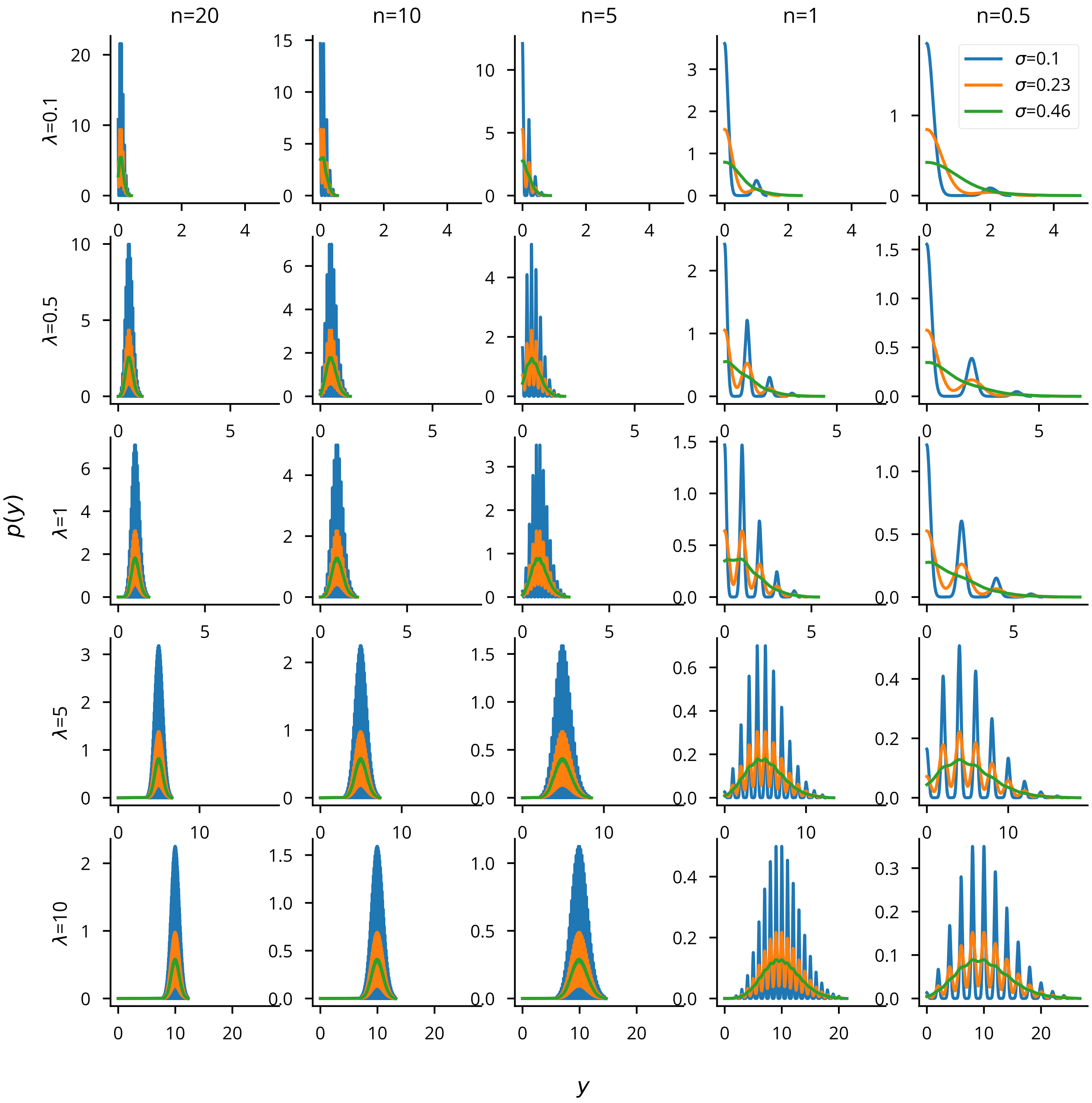


Figure S7: A visualization to assess the appropriateness of the Gaussian noise model for imaging experiments. We plot the Gaussian distribution $p(y) = \mathcal{N}(x, \sigma)$ over a Poisson photon count per pixel $x \sim \text{Pois}(\lambda)$. $N$ refers to the number of pixels visible in the ROI and $\lambda$ is the expected photon count. Plots show a roughly Gaussian-distributed $p(y)$ when $N > 1$, which is a realistic assumption for imaging experiments. The spikiness would be mitigated in a real experiment, where $\lambda$ and $\sigma$ would not be constant across pixels. The Gaussian observation appears to be least appropriate for low photon counts, where the distribution has a heavy right tail.

bioRxiv preprint doi: https://doi.org/10.1101/2023.01.27.525963; this version posted August 23, 2025. The copyright holder for this preprint (which was not certified by peer review) is the author/funder, who has granted bioRxiv a license to display the preprint in perpetuity. It is made available under aCC-BY 4.0 International license.

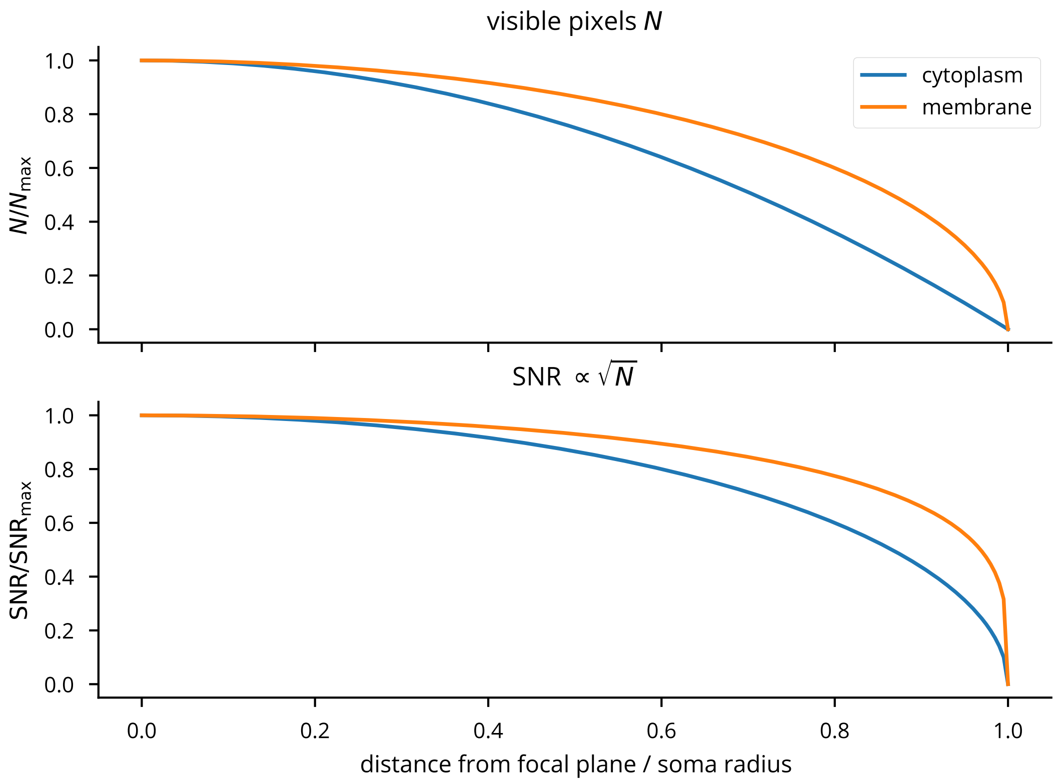


Figure S8: Plot of the number of visible pixels $N$ and the SNR as a function of the distance from the focal plane, for indicators found both in the cytoplasm (calcium indicators) and membrane (voltage indicators).

bioRxiv preprint doi: https://doi.org/10.1101/2023.01.27.525963; this version posted August 23, 2025. The copyright holder for this preprint (which was not certified by peer review) is the author/funder, who has granted bioRxiv a license to display the preprint in perpetuity. It is made available under aCC-BY 4.0 International license.

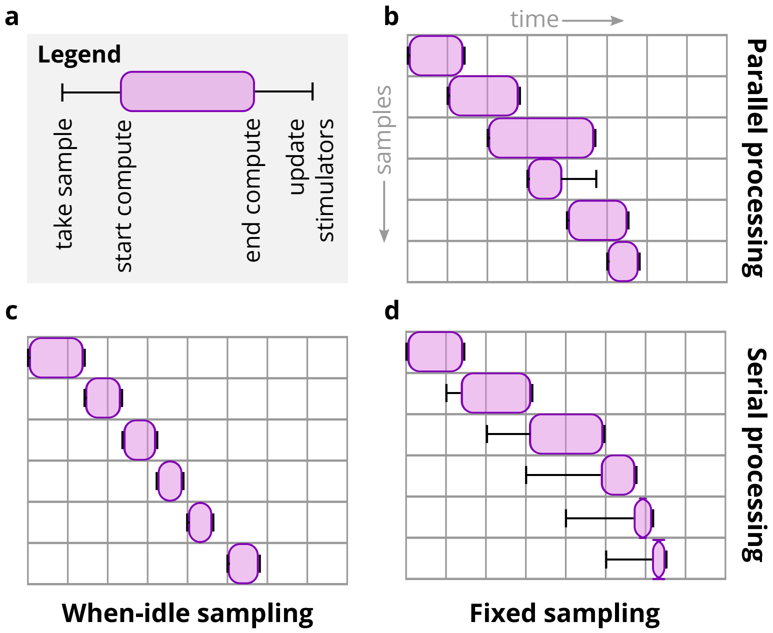


Figure S9: Latency emulation strategy and available configurations. (A) Cleo registers the time a sample is taken from the recording devices, determines the times the computation starts and ends, applies the user-specified delay, and updates stimulation devices when finished. (B) The default parallel processing/fixed sampling mode. Updates are reserved until the previous update is delivered so the sequence of stimulator updates corresponds to the sequence of measurements. (C) The "when-idle" processing mode samples only once the computation for the previous step has terminated. (D) The serial processing/fixed sampling case reflects when computations are not performed in parallel, but sampling continues on a fixed schedule. Samples are taken either as soon as possible after the previous sample time was missed, or on schedule otherwise.

bioRxiv preprint doi: https://doi.org/10.1101/2023.01.27.525963; this version posted August 23, 2025. The copyright holder for this preprint (which was not certified by peer review) is the author/funder, who has granted bioRxiv a license to display the preprint in perpetuity. It is made available under aCC-BY 4.0 International license.

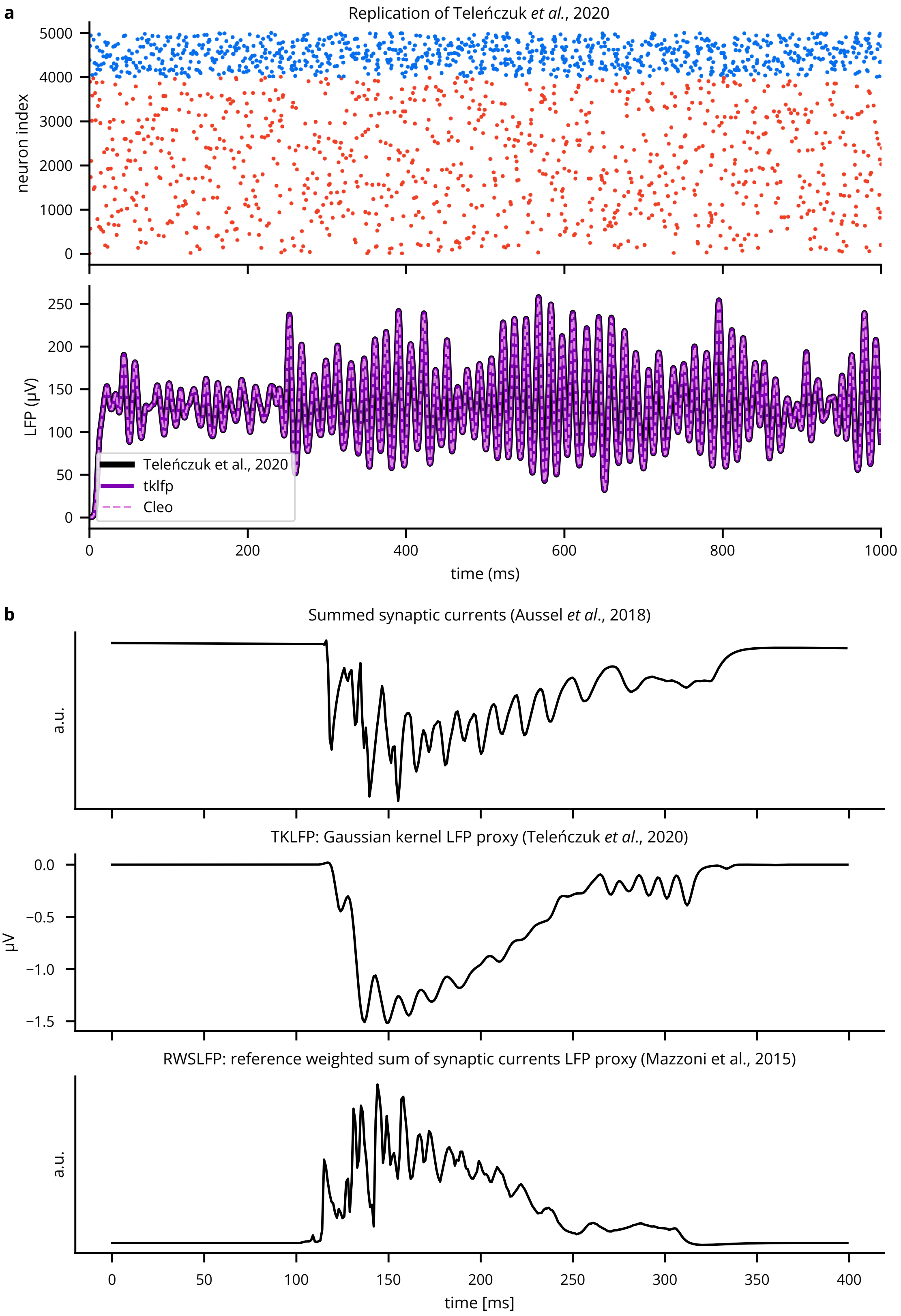


Figure S10: Verification of LFP proxy methods. (A) Replication of the Teleńczuk kernel LFP demo (Telenczuk et al., 2020). (B) Comparison of LFP proxy signals during SWR-like activity in a hippocampus model (see Sec. 3.3.3). Aussel et al. (2018) represent LFP with a sum of synaptic currents, each neuron's contribution depending on its location in space. The Gaussian kernel approximation method is as described by Telenczuk et al. (2020) and computed by Cleo, which uses the `tklfp` package implementation (Johnsen, 2022). The reference weighted sum method is described by Mazzoni et al. (2015) and is also computed by Cleo, which uses the `wslfp` package implementation (Johnsen et al., 2024) (see Sec. 2.4.2).

bioRxiv preprint doi: https://doi.org/10.1101/2023.01.27.525963; this version posted August 23, 2025. The copyright holder for this preprint (which was not certified by peer review) is the author/funder, who has granted bioRxiv a license to display the preprint in perpetuity. It is made available under aCC-BY 4.0 International license.

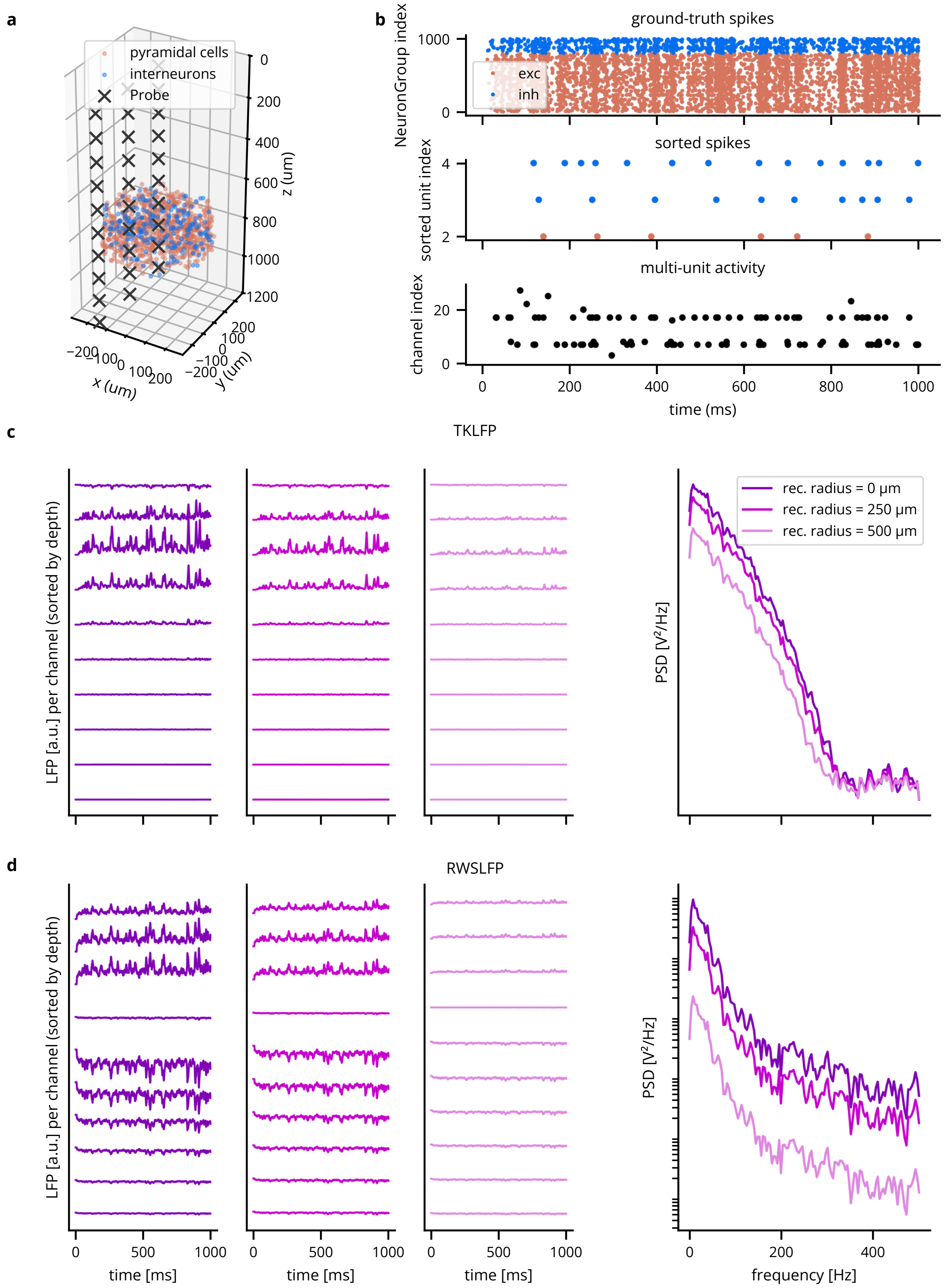


Figure S11: Comparison of the TKLFP and RWSLFP proxy methods for a simulated E/I network. We see here that TKLFP captures less high-frequency content, which is as reported by Teleńczuk *et al.* (A) A Cleo-generated plot of the network model and electrode placement. (B) Sorted and multi-unit spiking activity recorded from the network. (C) LFP and power spectral density (PSD) for the TKLFP signal recorded by the electrode. (D) Same as *C*, but for the RWSLFP signal.
