# Supplementary material for: Bridging model and experiment in systems neuroscience with Cleo: the Closed-Loop, Electrophysiology, and Optophysiology simulation testbed
Source: bioRxiv. 2024 Jul 9:2023.01.27.525963. Preprint. [Version 2] doi: 10.1101/2023.01.27.525963 (PMC11257437; doi:10.1101/2023.01.27.525963)
Supplement: Supplementary file 1 [file media-1.pdf]

Supplementary Information for "Bridging model and experiment in systems neuroscience with Cleo: the Closed-Loop, Electrophysiology, and Optophysiology simulation testbed"

1. Multi-wavelength sensitivity

1.1. Light superposition

Here we describe how we model dynamics for light of two different wavelengths, given photon flux ϕ_{λ_1} and ϕ_{λ_2}. Bansal *et al.* [1] use a weighted sum of activation functions

$$G(\phi) = G(\phi_{\lambda_1}) + \varepsilon G(\phi_{\lambda_2}).$$

However, these are sublinear functions, so adding them results in exaggerated activation. In the extreme case, imagine two light sources that are just 1 nm apart in wavelength, with $\varepsilon = 1$. Thus:

$$\begin{gathered} \phi_{\lambda_1} = \phi_{\lambda_2} = \phi_{tot}/2 \\ G(\phi_{tot}) \approx G(\phi_{\lambda_1}) + G(\phi_{\lambda_2}) = 2G(\phi_{tot}/2), \end{gathered}$$

which contradicts what we expect for sublinear G:

$$G(\phi) < 2G(\phi/2)$$

A more accurate approach instead assumes the following

$$G(\varphi_{\lambda_2}) = \varepsilon G(\phi_{\lambda_2}),$$

where φ_{λ_2} is the standard-wavelength equivalent ("effective flux") of ϕ_{λ_2}.

Demonstrating with G_{a1} of the four-state model [2], we solve for φ in terms of ϕ and ε:

$$\begin{aligned} G_{a1}(\varphi) &= \varepsilon G_{a1}(\phi) \\ \frac{\varphi^p}{\varphi^p + \phi_m^p} &= \varepsilon \frac{\phi^p}{\phi^p + \phi_m^p} \\ \varphi^p \phi^p + \varphi^p \phi_m^p &= \varepsilon(\varphi^p + \phi_m^p)\phi^p \\ (\phi^p + \phi_m^p - \varepsilon\phi^p)\,\varphi^p &= \varepsilon\phi^p\phi_m^p \\ \varphi^p &= \frac{\varepsilon\phi^p\phi_m^p}{(1-\varepsilon)\phi^p + \phi_m^p} \\ \varphi &= \left(\frac{\varepsilon\phi^p\phi_m^p}{(1-\varepsilon)\phi^p + \phi_m^p}\right)^{1/p}. \end{aligned}$$

We then compute our activation functions as

$$G(\phi_{\lambda_1}, \phi_{\lambda_2}) = G(\phi_{\lambda_1} + \varphi_{\lambda_2}).$$

Unfortunately, this doesn't yield a simple constant conversion factor. However, if we approximate G as linear, we can use a weighted sum of fluxes:

$$G(\phi) = G(\phi_{\lambda_1} + \varepsilon\phi_{\lambda_2})$$

Plotting G for multiple opsins shows this linear approximation does yield a lower activation curve than the Bansal *et al.* approach and is qualitatively close to the true φ derived above. See Extended Data Fig. **??** for a comparison of the three methods.

1.2. Action spectrum normalization

Some action spectra are measured with equal power density/pulse width across wavelengths, while others are reported with equal photon flux. We store ours as equal power density spectra since they seem to be more common and allow for the opsin model to use both accurate power density and photon flux values. We use ε to represent sensitivity relative to the peak-sensitivity wavelength, ε_ϕ and ε_P representing the equal photon flux and power density versions, respectively.

For a given wavelength λ,

$$\varepsilon(\lambda) = \frac{G(\varphi_\lambda)}{G(\phi)} = \frac{G_\lambda}{G(\phi)}.$$

We let G_λ represent the response at wavelength λ, while $G(\phi)$ represents the response at the peak wavelength for the same flux ϕ. We will assume G is a linear function, as above, using C to represent a constant:

$$\varepsilon_\phi(\lambda) = \frac{G_\lambda}{G(\phi)} \qquad \varepsilon_P(\lambda) = \frac{G_\lambda}{G(\phi)}$$
$$\varepsilon_\phi(\lambda) = \frac{G_\lambda}{C\phi} \qquad \varepsilon_P(\lambda) = \frac{G_\lambda}{C\phi}$$

Then we make either photon flux or irradiance (power density) constant:

$$\varepsilon_\phi(\lambda) = \frac{G_\lambda}{C\phi_{\text{const}}} \qquad \varepsilon_P(\lambda) = \frac{G_\lambda}{CI_{\text{const}}/e_{\text{photon}}}$$
$$\varepsilon_P(\lambda) = \frac{G_\lambda}{CI_{\text{const}}\lambda}$$
$$\varepsilon_\phi(\lambda) = \frac{G_\lambda}{C} \qquad \varepsilon_P(\lambda) = \frac{G_\lambda}{C\lambda}.$$

$$C\varepsilon_\phi(\lambda) = C\lambda\varepsilon_P(\lambda) = G_\lambda$$
$$\varepsilon_P(\lambda) = C\frac{\varepsilon_\phi(\lambda)}{\lambda}$$

Thus, we can convert from $\varepsilon_\phi(\lambda)$ to $\varepsilon_P(\lambda)$ by dividing by λ and normalizing.

2. GECI convolution simulation as an ODE

Song *et al.* convolve the intracellular calcium trace $\left[\text{Ca}^{2+}\right]$ with a double exponential kernel to capture variable rise and decay times in the fluorescence signal. To simplify simulation (to not have to keep a buffer of past calcium values), we can represent this convolution as an ODE. Let $c(t)$ and $b(t)$ be the free and bound calcium concentrations and $h(t)$ be the kernel function

$$h(t) = u(t)A\left(1 - e^{-t/\tau_{\text{on}}}\right)e^{-t/\tau_{\text{off}}}$$
$$b(t) = c(t) * h(t),$$

where $u(t)$ is the unit step function, included to ensure the kernel is causal.

We can represent the convolution as multiplication in the Laplace domain:

$$B(s) = C(s)H(s).$$

By expanding out $h(t)$, we get functions that can easily be transformed into the Laplace domain. Let $\kappa = \tau_{\text{off}}^{-1}, \lambda = \tau_{\text{off}}^{-1} + \tau_{\text{on}}^{-1}$ to simplify notation.

$$\begin{aligned} h(t) &= Au(t)\left(1 - e^{-T_{\text{on}}t}\right)e^{-T_{\text{off}}t} \\ &= A(u(t)e^{-\kappa t} - u(t)e^{-\lambda t}) \\ H(s) &= A\left(\frac{1}{s+\kappa} - \frac{1}{s+\lambda}\right) \\ B(s) &= A\left(\frac{1}{s+\kappa} - \frac{1}{s+\lambda}\right)C(s). \end{aligned}$$

Now we get a common denominator and rearrange:

$$\begin{aligned} B(s) &= A\left(\frac{\lambda - \kappa}{(s+\kappa)(s+\lambda)}\right)C(s) \\ B(s) &= A\left(\frac{\lambda - \kappa}{s^2 + (\kappa+\lambda)s + \kappa\lambda}\right)C(s) \\ \left(s^2 + (\kappa+\lambda)s + \kappa\lambda\right)B(s) &= A\left(\lambda - \kappa\right)C(s) \end{aligned}$$

Now we use the s^2 and s terms to convert to a second-order ODE, using the fact that the Laplace transform of $b''(t)$ and $b'(t)$ are $s^2B(s) - sb(0^-) - b(0^-)$ and $sB(s) - b(0^-)$, respectively. Also, we assume that $b(0) = b'(0) = 0$ to avoid undefined $\delta(t)$ and $\delta'(t)$ terms after the inverse Laplace transform; this appears to have only a minor effect.

$$b''(t) + (\kappa+\lambda)b'(t) + \kappa\lambda b(t) = A(\lambda - \kappa)c(t).$$

Rearranging to a first-order ODE system by introducing $\beta(t) = b'(t)$, we get

$$\begin{aligned} b'(t) &= \beta(t) \\ b''(t) &= \beta'(t) = A(\lambda-\kappa)c(t) - (\kappa+\lambda)\beta(t) - \kappa\lambda b(t). \end{aligned}$$

Special thanks to DinosaurEgg on Math Stack Exchange for helping solve this problem.

3. Hippocampal epilepsy model validation

Theta band power was computed using SciPy's `spectrogram` function with a Tukey window of width 3.906 sec, $\alpha = 0.25$, and overlap width of 3.809 sec.

4. Prospective experiment 3

The reference signal was generated by delivering a 1 nA square wave input from 100 to 300 ms to entorhinal cortex using the original model's I_{ext} term, without noise added. Training data was generated by running the system for 13 seconds with alternating on and off periods of length $T \sim \mathcal{N}(200,\ 50)$ µs. During "on" intervals, $I_{\text{ext}} \sim |\mathcal{N}(0, \text{Irr}_{\text{max}}/3)|$. Irr_{max} was 75 mW/mm^2, which is described as an upper safety limit for 473 nm light delivery to the brain [3]. Gaussian process noise was generated with mean $\mu = 0.167$ nA and using the exponentiated quadratic kernel

$$k(t_1, t_2) = \sigma^2 e^{\frac{-(t_2-t_1)^2}{2l^2}} \tag{1}$$

with $\sigma = 0.083$ nA, $l = 30$ ms and was added to input current I_{ext} in each control scenario. The parameters of light delivery were altered from the 473 nm optic fiber defaults to allow for greater propagation—K and S were both divided by 10. The training data was fit using the ldsCtrlEst library's SSID and EM fitting methods with latent dimensionality $n_x = 4$ [4]. ldsCtrlEst's Gaussian linear quadratic regulator (LQR)

controller was used with a gain computed from $Q = C^T C$, $R = 0.001$ state and input penalties, with a simulated 3 ms of latency.

Model-predictive control (MPC) was implemented with a control and prediction horizon of 6 and 36 control periods (each of which was 3 ms long) respectively. The standard, quadratic cost function utilized constant error weights $Q = C^T C$, $R = 10^{-6}$, and was optimized using OSQP via the JuMP optimization interface [5, 6]. MPC was simulated with 6 ms latency.

References

[1] H. Bansal, N. Gupta, S. Roy, Theoretical Analysis of Low-power Bidirectional Optogenetic Control of High-frequency Neural Codes with Single Spike Resolution, Neuroscience 449 (2020) 165–188. doi:10.1016/j.neuroscience.2020.09.022.
URL https://www.ibroneuroscience.org/article/S0306-4522(20)30596-0/fulltext 1

[2] B. D. Evans, S. Jarvis, S. R. Schultz, K. Nikolic, PyRhO: A Multiscale Optogenetics Simulation Platform, Frontiers in Neuroinformatics 10 (MAR) (2016) 8. doi:10.3389/fninf.2016.00008.
URL http://journal.frontiersin.org/Article/10.3389/fninf.2016.00008/abstract 1

[3] J. A. Cardin, M. Carlén, K. Meletis, U. Knoblich, F. Zhang, K. Deisseroth, L. H. Tsai, C. I. Moore, Targeted optogenetic stimulation and recording of neurons in vivo using cell-type-specific expression of Channelrhodopsin-2, Nature Protocols 5 (2) (2010) 247–254. doi:10.1038/nprot.2009.228.
URL https://www.nature.com/articles/nprot.2009.228 3

[4] M. F. Bolus, A. A. Willats, K. A. Johnsen, LDS Control & Estimation (Aug. 2022).
URL https://cloctools.github.io/lds-ctrl-est/ 3

[5] B. Stellato, G. Banjac, P. Goulart, A. Bemporad, S. Boyd, OSQP: An operator splitting solver for quadratic programs, Math. Prog. Comp. 12 (4) (2020) 637–672. doi:10.1007/s12532-020-00179-2.
URL http://link.springer.com/10.1007/s12532-020-00179-2 4

[6] M. Lubin, O. Dowson, J. D. Garcia, J. Huchette, B. Legat, J. P. Vielma, JuMP 1.0: Recent improvements to a modeling language for mathematical optimization (Mar. 2023). arXiv:2206.03866, doi:10.48550/arXiv.2206.03866.
URL http://arxiv.org/abs/2206.03866 4
